# Investigation of rare and low-frequency variants using high-throughput sequencing with pooled DNA samples

**DOI:** 10.1038/srep33256

**Published:** 2016-09-16

**Authors:** Jingwen Wang, Tiina Skoog, Elisabet Einarsdottir, Tea Kaartokallio, Hannele Laivuori, Anna Grauers, Paul Gerdhem, Marjo Hytönen, Hannes Lohi, Juha Kere, Hong Jiao

**Affiliations:** 1 Department of Biosciences and Nutrition, Karolinska Institutet, SE-14183 Huddinge, Sweden; 2 Science for Life Laboratory, Stockholm, Sweden; 3 Molecular Neurology Research Program, University of Helsinki and Folkhälsan Institute of Genetics, Helsinki, Finland; 4 Medical and Clinical Genetics, University of Helsinki and Helsinki University Hospital, Helsinki, Finland; 5 Obstetrics and Gynecology, University of Helsinki and Helsinki University Hospital, Helsinki, Finland; 6 Institute for Molecular Medicine Finland, University of Helsinki, Helsinki, Finland; 7 Department of Orthopedics, Karolinska University Hospital and Department of Clinical Sciences, Intervention and Technology (CLINTEC) Karolinska Institutet, Stockholm, Sweden; 8 Department of Orthopaedics, Sundsvall and Harnosand County Hospital, Sundsvall, Sweden; 9 Department of Veterinary Biosciences, and Research Programs Unit, Molecular Neurology, University of Helsinki and Folkhälsan Research Center, Helsinki, Finland

## Abstract

High-throughput sequencing using pooled DNA samples can facilitate genome-wide studies on rare and low-frequency variants in a large population. Some major questions concerning the pooling sequencing strategy are whether rare and low-frequency variants can be detected reliably, and whether estimated minor allele frequencies (MAFs) can represent the actual values obtained from individually genotyped samples. In this study, we evaluated MAF estimates using three variant detection tools with two sets of pooled whole exome sequencing (WES) and one set of pooled whole genome sequencing (WGS) data. Both GATK and Freebayes displayed high sensitivity, specificity and accuracy when detecting rare or low-frequency variants. For the WGS study, 56% of the low-frequency variants in Illumina array have identical MAFs and 26% have one allele difference between sequencing and individual genotyping data. The MAF estimates from WGS correlated well (*r* = 0.94) with those from Illumina arrays. The MAFs from the pooled WES data also showed high concordance (*r* = 0.88) with those from the individual genotyping data. In conclusion, the MAFs estimated from pooled DNA sequencing data reflect the MAFs in individually genotyped samples well. The pooling strategy can thus be a rapid and cost-effective approach for the initial screening in large-scale association studies.

In the last two decades, more than 10,000 variants associated with complex traits have been identified by genome-wide association studies (GWAS)^1^. However, most of the target sites of GWAS have been common variants (risk allele frequency >5%) with modest or weak genetic effects, usually requiring large sample sizes for detection at the genome-wide significant level^2^. On the other hand, it is possible that common diseases are partially caused by rare and generally deleterious variants with a strong impact on the risk of disease in individual patients^3^. The majority of those low-frequency variants have not been investigated by single-nucleotide polymorphism (SNP) array-based GWAS, as the arrays primarily target common variants.

High-throughput next generation sequencing (NGS) technologies have revolutionised genetic research by enabling the identification of rare and low-frequency genetic variation on a massive scale^4^,^5^. In contrast to SNP array genotyping, next generation DNA sequencing does not rely on pre-designed probes against target sequences and is therefore able to detect any variant within the studied genome. Moreover, the new technology greatly reduces per base pair sequencing cost, provides high read coverage and depth and produces an abundance of sequencing reads at both the whole genome and exome wide scale. It has contributed to the mapping of a number of genetic variants underlying Mendelian diseases^6^,^7^,^8^, and there is strong interest in extending the application of NGS to complex traits^9^.

Whole genome sequencing (WGS) and whole exome sequencing (WES)^10^,^11^ are becoming increasingly popular because of their wide coverage and single-base resolution. However, these techniques are still costly, laborious and time-consuming for most laboratories involved in population-based association studies. To capture rare variants related to complex diseases, the ideal approach is to sequence every individual sample in a very large cohort^12^. An alternative approach is to pool a number of individual DNA samples and sequence the pooled DNA, which can efficiently increase sample size and sequencing depth at a reduced cost and effort in library preparation. In addition to enabling the identification of rare variants in candidate genes^13^,^14^, certain pooled DNA sequencing studies at the whole exome scale have also reported low-frequency variants associated with complex diseases^15^,^16^.

The essential question using a pooling strategy for genetic studies focused on rare and low-frequency variants includes two aspects: (1) whether the variants can be detected reliably and (2) whether minor allele frequencies (MAFs) can be estimated accurately using pooled samples. Here, we report the evaluation of the pooling strategy based on WES data from studies on human idiopathic scoliosis^17^ and pre-eclampsia, as well as on WGS data from a study on canine compulsive tail-chasing behaviour. We compared the variants detected by three different tools (SAMtools, GATK and Freebayes) and evaluated the accuracy of MAF estimates from pooled DNA sequencing data by comparing them with the MAFs obtained from individual genotyping data.

## Results

### Pooled whole exome sequencing

#### Read alignment and single nucleotide variant (SNV) detection

The procedure of sequencing read analysis is illustrated in Supplementary Fig. S1. In the scoliosis study, we obtained 307 to 412 million sequence reads in each sample pool. Of the sequence reads, 93.3% to 98.5% could be mapped to the human reference genome (GRCh37/hg19) (Supplementary Table S1). After removing PCR duplicates, the average depth of mapped reads in each pool ranged from 104x to 194x. Approximately 40–46 Mb (77–89%) of the SureSelect enrichment regions were covered over 30x and 29–41 Mb (56–79%) were covered over 60x, with 0.7–2% of regions not covered (Supplementary Table S2). In the pre-eclampsia study, WES generated 290 to 487 million reads per pool and over 98% of the sequencing reads could be mapped to the human reference genome (GRCh37/hg19) (Supplementary Table S1). After removing duplicates, the average read depth of the enrichment regions was over 200x in all but two pools (95x in pool 1 and 169x in pool 10). Over 99% of the enrichment regions were covered. Except for the two pools with a high duplicate rate (pool 1 and pool 10), at least 46 Mb (90%) of the target regions were covered over 30x and over 42 Mb (82%) were covered over 60x (Supplementary Table S2).

Taking uniquely mapped reads as input, we detected single nucleotide variants (SNVs) in WES data using three tools, SAMtools, GATK and Freebayes, with different ploidy settings. At first, we compared the SNVs detected using two different GATK UnifiedGenotyper ploidy settings, assuming diploidy or ploidy of 20. Supplementary Fig. S2 indicates that the SNVs detected by GATK using the ploidy setting of 20 covered most of the SNVs identified by GATK with the default diploid setting. Accordingly, we applied a ploidy setting of 20 when using the GATK UnifiedGenotyper programme in the subsequent detection of SNVs in the WGS and WES pooled data. SAMtools, GATK and Freebayes detected over 2 million SNVs in the scoliosis pools and 2.4 to 3.2 million SNVs in the pre-eclampsia pools (Supplementary Table S3). In both WES studies, we obtained the largest number of SNVs (2.2 million in scoliosis and over 3.2 million in pre-eclampsia) using GATK. Compared with the other two tools, a higher proportion of the SNVs detected by Freebayes were singletons (SNVs in only one pool). Compared with GATK and Freebayes, SAMtools provided fewer rare variants, but more of the common variants. However, most of the common variants uniquely identified by SAMtools were located outside the Agilent SureSelect kit enrichment regions and were based on very low sequence coverage data.

#### Comparisons of SNVs from three variant detection tools

**Figure 1:**
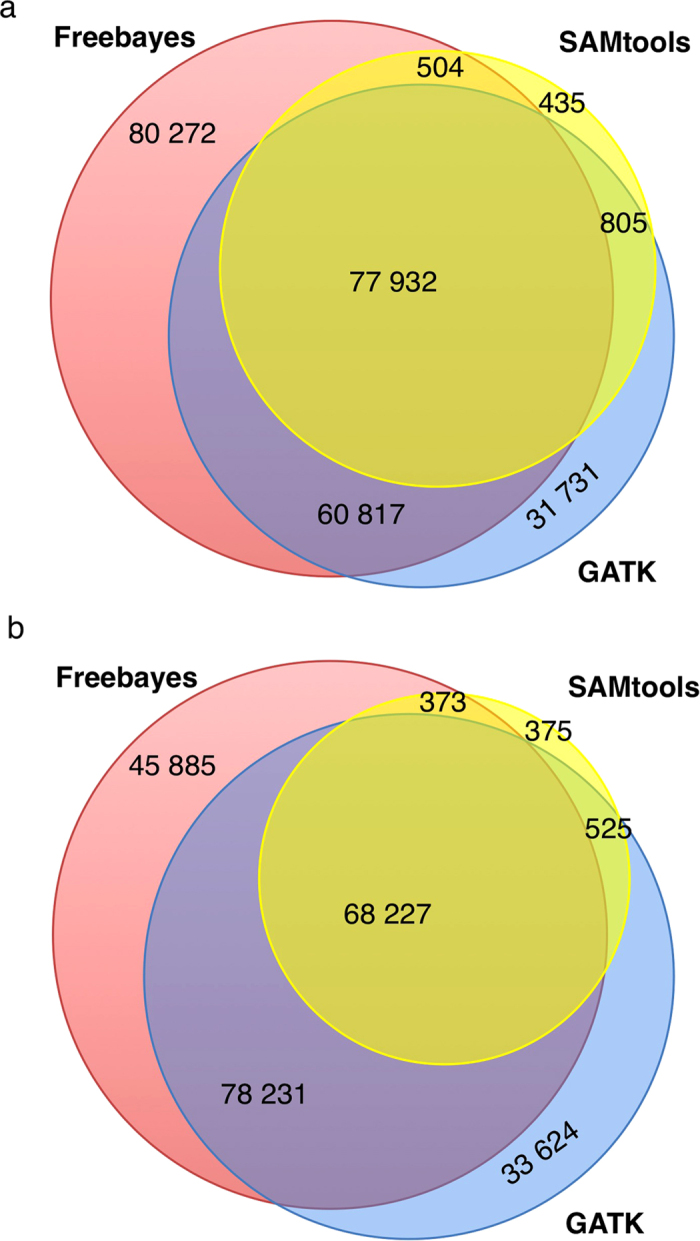
Comparison of SNV detection by SAMtools, GATK (ploidy setting) and Freebayes (ploidy setting) in the WES studies. The blue, red and yellow circles represent the SNVs detected by GATK UnifiedGenotyper (ploidy setting), Freebayes (ploidy setting) and SAMtools, rescpectively. (**a**) The SNVs in the Agilent SureSelect target regions in the scoliosis study. (**b**) The SNVs in the Agilent SureSelect target regions in the pre-eclampsia study.

When retaining the SNVs located in the Agilent SureSelect kit enrichment regions, the number of SNVs detected by all three tools was 77,932 in the scoliosis and 68,227 in the pre-eclampsia studies (Fig. 1). Freebayes detected the largest number of SNVs in the enrichment regions. By contrast, SAMtools detected the fewest SNVs and the majority of these SNVs (99%) were also detected by GATK.

As the overlap of detected SNVs showed a similar pattern in both WES studies (Fig. 1), we selected the scoliosis study as an example to describe the variant detection performance of the three tools, investigating the SNVs within the enrichment regions. Both GATK and Freebayes detected more than twice as many SNVs as SAMtools did (Fig. 1a). The difference in the numbers of SNVs was due to novel variants that were not annotated in dbSNP, or rare variants with alternative allele frequency (AAF) less than 1%. In particular, 93% of the SAMtools-detected and 84% of the GATK-detected SNVs were already annotated in dbSNP 144, but the annotation rate dropped to 61% with Freebayes. When the detected SNVs were grouped by AAFs, the three tools provided a comparable amount of common (AAF > 5%) SNVs, while the amount of rare (AAF < 1%) and low-frequency (1% ≤ AAF ≤ 5%) SNVs varied greatly among the tools (Table 1). Moreover, around half of the SNVs detected by Freebayes did not have alternative allele frequency information in the 1000 Genomes project database, in contrast to this proportion being only 10% for the SAMtools-detected SNVs (Table 1). When classifying the SNVs based on the number of pools they were detected in, we saw that both SAMtools and GATK detected fewer singletons (14–19%) than Freebayes (43%, Supplementary Fig. S3).

Because of the high detection rate of potential novel and rare SNVs by Freebayes, we further investigated the 80,272 Freebayes-specific SNVs in the enrichment regions (Supplementary Fig. S4). Less than half of the SNVs were supported by at least 60x reads and over 20% were covered by less than 30x read depth. Over 90% of the SNVs were identified in only one scoliosis pool or did not have AAF information in the 1000 Genomes project (Supplementary Fig. S4). When we set filtering for variant quality score, 96% of the Freebayes-specific SNVs had a quality score of less than 3, i.e. less than 50% probability of being truly polymorphic. Only 2% of them had over 90% probability of being polymorphic, (quality score more than 10), suggesting that the majority of Freebayes-specific SNVs were likely false positives.

#### Genotyping validation

We selected 50 and 59 SNVs from the scoliosis and pre-eclampsia WES studies, respectively, for validation by using genotyping (Supplementary Table S4). The same 100 DNA samples utilised in each WES study were genotyped individually using the Sequenom MassARRAY system. We successfully validated 42 scoliosis and 44 pre-eclampsia related SNVs after excluding four monomorphic sites (one in the scoliosis and three in the pre-eclampsia studies). The four false positive SNVs were all annotated in dbSNP 144, but none of them had MAF information in the 1000 Genomes project database. In total, 95.6% of the selected SNVs were validated in each dataset.

All four false-positive SNVs were identified as polymorphic by GATK, and Freebayes also identified the three SNVs in the pre-eclampsia study as polymorphic. SAMtools had the lowest false-positive detection, identifying three of the variants as monomorphic, while it missed several loci that were actually polymorphic in both studies (Supplementary Fig. S5).

#### Evaluation of MAF estimates

**Figure 2:**
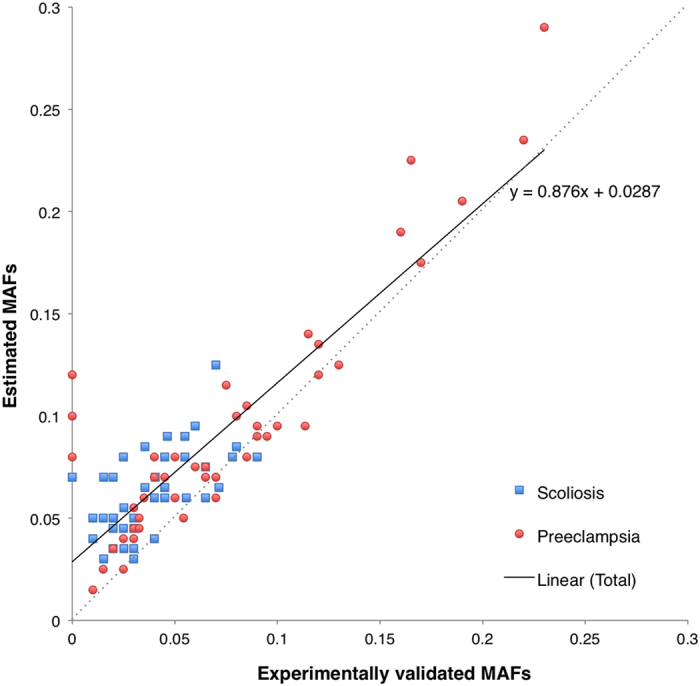
MAF comparison between the WES and the Sequenom genotyping. The estimated MAFs were estimated with GATK based on allele counts. The blue squares represent the 43 validated SNVs in the scoliosis study and the red dots the 47 validated SNVs in the pre-eclampsia study. The diagonal is shown with the grey dashed line.

Two strategies were applied for MAF estimates: using read depth and allele count. The concordance between the estimated and validated MAFs was measured with the root-mean-square deviation (RMSD). When using read depths for estimating MAF across all pools, most of the MAFs in the pooled samples were enlarged but the best-fit trend line of the SAMtools estimation was more diagonal than those of the other two tools. However, the MAFs estimated based on read depths with three tools varied a lot (Supplementary Table S5). When using allele counts for estimating MAF, both GATK and Freebayes showed similar accuracies of the estimated MAFs (RMSD = 0.031–0.032). The MAFs estimated using allele counts with GATK from the pooled DNA sequencing data showed high concordance with those from the individual genotypes (Pearson correlation coefficient, *r* = 0.88) (Fig. 2) even though they were slightly overestimated in the WES data.

As the SNVs validated in the pre-eclampsia dataset included rare, low-frequency and common variants, we selected the 47 SNVs in the pre-eclampsia study to evaluate the GATK-estimated MAFs of all SNVs in each individual pool, as well as the GATK-estimated MAFs of each SNV in all pools. The comparisons of MAFs based on each pool (Supplementary Fig. S6) revealed that SNVs in two of the pools (6 and 9) showed higher concordance between the estimated and validated MAFs than those in other pools. For more than half of the validated SNVs, the MAFs were overestimated by using WES data. The estimated MAFs of some SNVs had a larger difference from the actual MAFs (more than two times) in some pools (Supplementary Fig. S7).

To investigate the effect of number of sequencing reads (i.e., depth of sequencing) on variant detection and MAF estimation, we randomly selected 70% and 80% of the reads from pre-eclampsia pool 7 and detected SNVs using GATK on just the pool 7 with the selected reads. Among the three false positive SNVs that validated to be monomorphic, all of them were identified as variants when using randomly selected 70% and 80% of total reads. To evaluate the accuracy of MAFs estimated from all reads and partial reads, MAFs were calculated based on allele counts. We took the average MAF of 10 rounds for both 70% and 80% of reads, and further compared them with the genotyping result for each SNV (Supplementary Fig. S8). A closer look at the MAF comparisons showed that when using partial reads for MAF estimation, each of 17 SNVs had the same MAF as the one obtained from total reads, whereas 8 SNVs showed smaller and over 20 SNVs with larger deviation from the validation when compared with the results based on 100% reads. The MAFs estimated using total reads demonstrated a higher concordance with the validation (*r* = 0.86), compared with those estimated using partial reads (*r* = 0.84).

**Figure 3:**
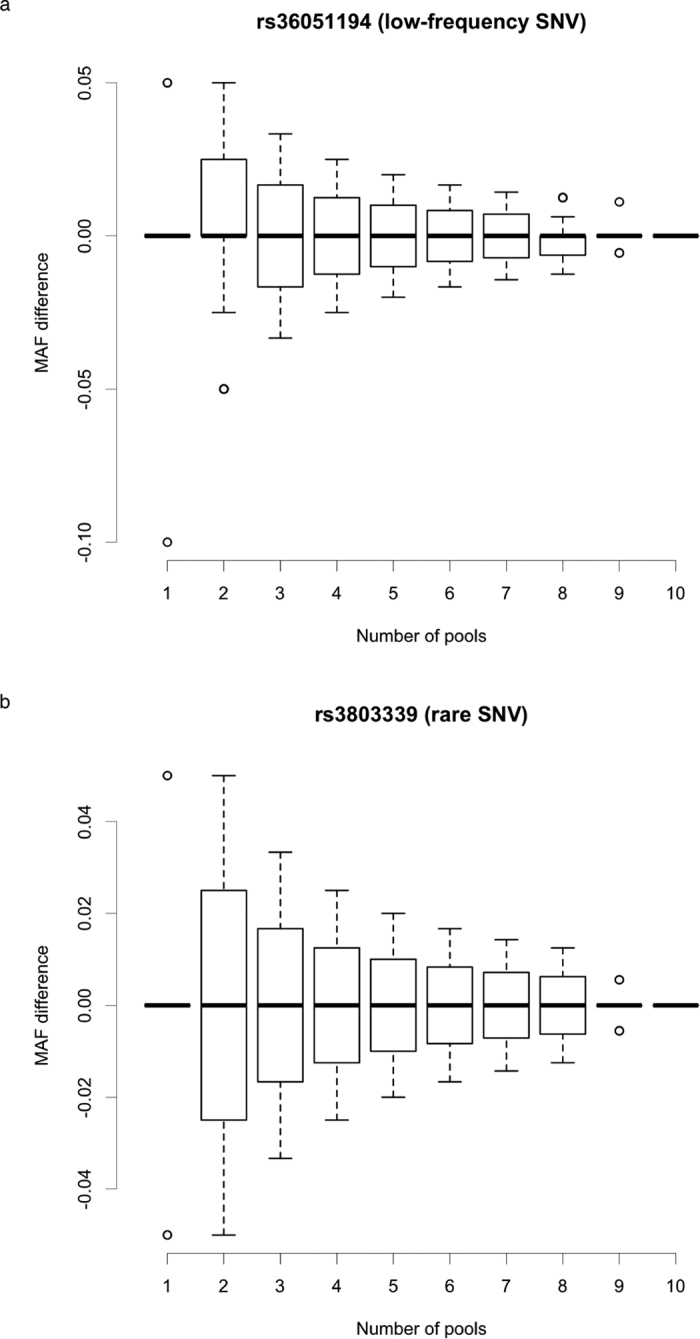
Distribution and variation of total MAFs estimated using a variable number of pre-eclampsia pools. The X-axis represents the number of pools used for estimating MAF from the exome sequencing data and the Y-axis shows the MAF difference between the exome sequencing estimation and the genotyping validation. (**a**) Low-frequency SNV rs36051194. (**b**) Rare SNV rs3803339.

A low-frequency SNV (rs36051194, Fig. 3a) and a rare SNV (rs3803339, Fig. 3b) were selected to demonstrate the effect of the number of pools used for variant detection on the accuracy of the MAF estimates. We randomly selected pre-eclampsia pools to calculate the difference between estimated and validated MAF values, starting from one to nine pools. The largest deviation from validated MAFs appeared when using single pool or two pools for MAF estimates based on allele counts in both SNVs. With the increase in the number of pools, the deviation tended to approach zero. Using more pools for variant detection resulted in more precise estimates of MAFs.

### Pooled whole genome sequencing

#### Canine SNP array

The 20 Bull Terriers in the WGS were previously genotyped as part of a study on canine compulsive tail-chasing behaviour, using Illumina Canine HD SNP arrays. The Canine HD array contained 172,371 markers in the canine genome. After filtering out those that failed quality control, 170,287 markers in the affected and 170,260 in the unaffected pool were further used for the evaluation of variant detection. Among those loci, 105,715 in the affected and 102,665 in the unaffected Bull Terriers were monomorphic. As the pooled samples contained both male and female Bull Terriers, we excluded the loci on sex chromosomes to reduce the complexity of MAF measurement. From the 166,813 autosomal markers left on the array, 1945 markers in the affected and 1972 markers in the unaffected Bull Terriers failed quality control. The remaining markers (164,868 in affected and 164,841 in unaffected dogs) were utilised as references for MAF comparison between the pooled DNA sequencing and the array genotyping.

#### Read alignment and SNV detection

The initial whole genome sequencing of pooled Bull Terrier DNA samples contained approximately 2.0 billion reads in each pool. Later, technical replicates with different libraries contained 1.4 billion and 1.7 billion reads in the affected and control pools, respectively. We combined two runs of sequencing and mapped the sequencing reads to the reference genome CanFam 3.1. After removing PCR duplicates, the mapping rates were 82–93% for total reads (Supplementary Table S1). The average depths were 133x in the affected samples pool and 135x in the control pool. Less than 1% of the genome was not covered at all, while over 97% of the genome was covered with at least 30x reads and 90–92% of the genome with at least 60x reads in both pools (Supplementary Table S2). With a ploidy setting of 20, GATK detected approximately 7.32 million SNVs in total, similar to the number of SNVs detected by Freebayes (7.61 million). By contrast, there were fewer SNVs (4.76 million) detected by SAMtools. About 88–92% of the SNVs detected were supported by at least 30x reads (Table 1).

#### Comparison of variant detection among the three tools

After removing the multi-allelic SNVs, the majority of SAMtools-generated SNVs (over 99%) were detected by GATK or Freebayes as well (Supplementary Fig. S9). The majority of SAMtools-specific SNVs (64%) were covered by reads less than 30x, while 75–95% of Freebayes and GATK-specific SNVs were covered by at least 30x reads. Approximately 30% of Freebayes-specific SNVs had over 50% probability of being polymorphic. However, as much as 92% of the rest of Freebayes-generated SNVs had over 50% probability of being polymorphic.

**Figure 4:**
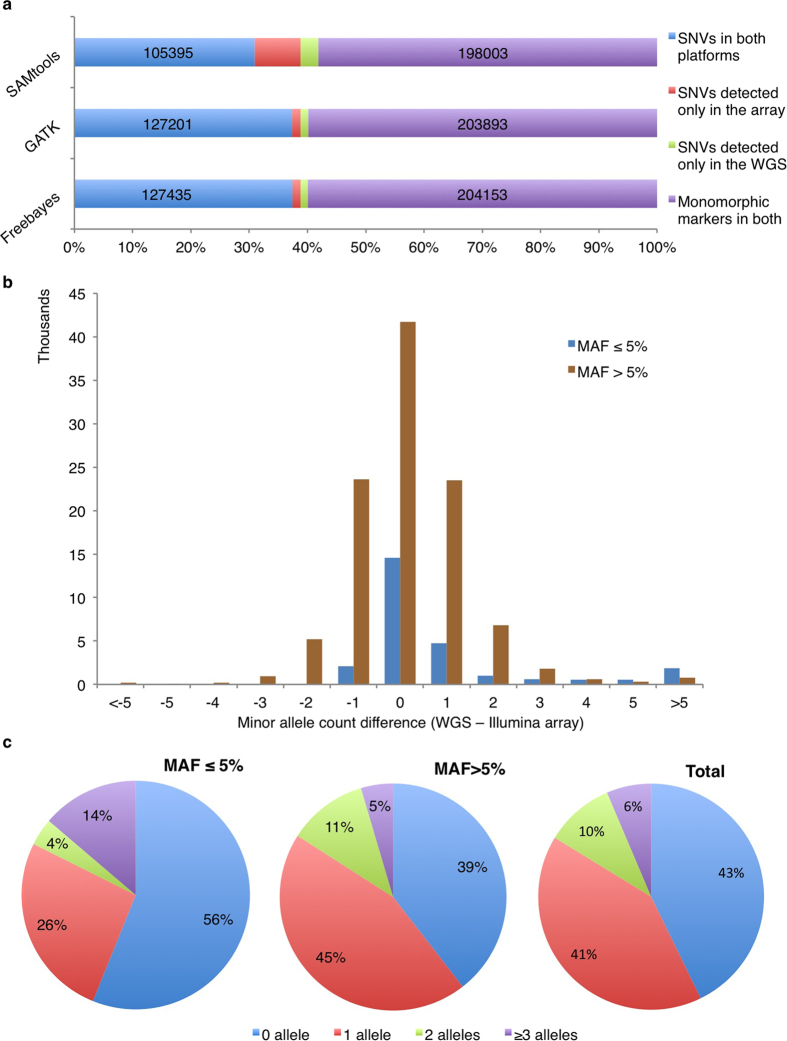
SNV detection across variant detection tools and minor allele count comparison between the WGS and the Illumina array in the bull terrier WGS study. (**a**) Polymorphic and monomorphic markers in the affected or the unaffected pool detected with the three variant detection tools and the Illumina array. (**b**) The variants in blue are SNVs with MAF < 5% in the genotyping data and the variants marked in brown are SNVs with MAF > 5% in the genotyping data. (**c**) Percentage of allele count difference (absolute value) between two platforms among polymorphic loci.

From the WGS data, GATK and Freebayes detected similar numbers of SNVs present in the Illumina array (Fig. 4a), and had the largest agreement on monomorphic markers. SAMtools missed over 26,000 SNVs (20.26% miss rate) that were detected in the array (Supplementary Table S6), while the miss rates of GATK or Freebayes detection were less than 4%. Furthermore, SAMtools detected more SNVs (3%) that were monomorphic in the array compared with GATK and Freebayes (1.2–1.3%). Therefore, using the Illumina array genotyping data as reference, the specificity, precision and accuracy of SAMtools detection were comparatively low (below 90%). By contrast, all measurements of the performance of GATK and Freebayes detection were over 96% (Table 2).

#### Evaluation of MAF estimates

Due to the high accuracy of variant detection, GATK was selected for estimating MAF from pooled sequencing data and for comparing MAFs between the WGS and the Illumina array data. When including monomorphic markers (60% of total) in both platforms, the concordance rate between the WGS and the arrays was 77% in two pooled samples (Supplementary Fig. S10). The Pearson correlation coefficient of the allele frequency of autosomal markers between the WGS and the Illumina arrays was 0.94. When the 198,162 autosomal monomorphic markers (100,415 in affected and 97,747 in unaffected samples) detected in both platforms were excluded, 56,260 SNVs (43%) had identical MAFs in both WGS and the Illumina array (Fig. 4b,c) and 53,870 SNVs (41%) had only one allele difference by direct allele counting. The concordance rate was even higher (56%) for the low-frequency variants with MAFs ≤5% in the array (Fig. 4c). The Pearson correlation coefficient of MAFs of the autosomal polymorphic markers between the WGS and the Illumina array was 0.85.

## Discussion

A large-scale sequencing project to discover rare and low-frequency variants that possibly contribute to disease is costly and time-consuming. It is advantageous to perform a pilot project that is fast and low-cost to explore highly interesting sequence variants that may occur among patients. This study was undertaken to test the feasibility of such an approach, i.e WGS and WES using pooled DNA. With this study, we discovered that (1) rare and low-frequency alleles were discovered with sufficiently high probability and (2) allele frequency estimates were sufficiently accurate. Our results suggest that a pooling approach that can cut costs using 10-plexed DNA samples may be a feasible choice as a piloting study in gene mapping projects. However, particular attention needs to be paid to read depth and the choice of variant detection tool.

In the experiments described here, sequencing was sub-optimal in one project, requiring a second round of WES because of the high duplicate rate in the scoliosis pools. Even though the total raw sequencing reads were similar in numbers in the scoliosis and pre-eclampsia studies, the final mapped and average read depths after duplicate removal differed considerably (Supplementary Table S1, S2). Re-running the sequencing with the same DNA library did not compensate for the loss caused by the high PCR duplicate rate. We nevertheless evaluated the influence of sequencing yields in order to test for the robustness of the pooling approach. As expected, fewer SNVs were detected in the WES pools with low read depth (Supplementary Table S7). However, the numbers of SNVs in the enrichment regions among pools were similar, with around 98.8–99.7% of total SNVs in the target regions. This suggests that our pooled sequencing was deep enough to discover the variants with average read depth at roughly 100x.

Among the three tested variant detection tools, SAMtools detected the lowest amount of SNVs, especially rare and low-frequency SNVs in the WES data. Moreover, in the comparisons of SNVs between WGS and Illumina array in the Bull Terrier study SAMtools exhibited lower sensitivity and specificity than the other two tools. Hence, SAMtools may not perform as well as GATK and Freebayes in rare variant detection. On the other hand, it worked well with detection and MAF estimates of common variants.

GATK and Freebayes detected more rare and low-frequency SNVs than SAMtools did, especially Freebayes identified large amount of singleton SNVs in the enrichment regions. The majority of Freebayes-specific SNVs were not annotated in the dbSNP database. The implausibly high proportion of unknown variants was also reported by another study^18^. Furthermore, almost all the Freebayes-specific SNVs had an extremely low variant detection quality, i.e. the probability of them being true polymorphic sites was very low. Consequently, quality filtering is critical for downstream processing of the SNVs detected with Freebayes. Even though there were a few false positive hits in GATK- and Freebayes-detected SNVs, both of the tools demonstrated high precision and accuracy in variant detection in general. Therefore, it may be advisable to identify rare and low-frequency variants in pooled DNA sequencing with one of these two tools. However, when using Freebayes, it is highly recommended to filter out the SNVs with low quality.

In general, the MAF estimates in pooled DNA sequencing were similar to those based on the individual genotyping data in all three studies (Figs 2 and 4). In particular, the large number of SNPs in the Illumina array gave us an opportunity to confirm the high accuracy of variant detection and high concordance of MAF estimates on a genome-wide scale. In the WES studies, the MAFs of low-frequency variants (AAF between 1% and 5%) tended to be overestimated in pooled sequencing (Supplementary Fig. S7). One potential cause might be one of the criteria of filtering SNVs for experimental validation i.e. the obtained SNVs had the largest difference between estimated MAFs and the MAFs in large population. Selecting random SNVs would probably have yielded to better accuracy, as shown in the bull terrier study. The effect could also be due to sampling, i.e. our sample size might have been too modest. We demonstrated that randomly reducing the read numbers in one pre-eclampsia pool led to larger deviation of MAF estimates in both directions in the selected pool (Supplementary Fig. S8). Moreover, the majority of the sites where the estimated MAFs differed considerably from the validated value tended to have lower read coverage, which indicated that sufficient read depth is critical for estimating allele frequency in pooled DNA sequencing.

Using the pooling layout setting in the two WES studies, we were able to detect SNVs with MAF of at least 0.5% in the 100 samples. However, as part of the filtering, we excluded singleton variants that appeared in only one pooled sample because it was not possible to distinguish a real rare variant from a technical error. Therefore the SNVs with MAFs less than 1% in the 100 samples were not selected for validation and association analysis. This decision caused the loss of detection of a number of rare variants that might be associated with phenotype. Therefore, when dealing with very rare variant, some cautions should be taken. For example, the unequal amount of DNA materials in a pool could affect the accuracy of MAF estimates, and poor or unequal coverage across pools could also influence the total MAF estimates. Evenly distributing each pool to every lane for sequencing could avoid such bias. Moreover, sequencing errors and misalignments of divergent short reads were difficult to identify, therefore an independent validation should be arranged. In addition, pooling reads do not provide individual haplotype information, which was needed for some gene burden tests.

In conclusion, by sequencing pooled DNA samples, we discovered a large amount of rare and low-frequency variants. The MAFs estimated from the pooled DNA sequencing data represented the MAFs from individual genotyping data with reasonable accuracy in our study. Our results demonstrated that the pooling strategy could be a cost-effective method as an initial screening procedure for rare and low-frequency variant association studies.

## Materials and Methods

### DNA sample collection and extraction

WES: A hundred Finnish women with pre-eclampsia were exome sequenced in 10 pools, each containing 10 DNA samples. Ninety of the study participants were selected from the Finnish Genetics of Pre-Eclampsia Consortium Cohort (FINNPEC). Ten women were from the pre-eclampsia family cohort used in previous linkage studies^19^,^20^. The genomic DNA was extracted from blood samples using a NucleoSpin Blood XL DNA extraction kit (Macherey-Nagel GmbH & Co.), a Chemagic Magnetic Separation Module I machine (Chemagen) or, in the case of the family samples, the phenol-chloroform method. The 100 genomic DNA samples were divided into 10 pools according to the sub-phenotypes of patients without using barcodes. The 10 family samples were pooled in a single pool. One hundred severe scoliosis patients from Sweden were subjected to exome sequencing with the same pooling strategy. The sample collection and DNA extraction have been previously described^17^. In both exome sequencing studies, 800 ng of each sample was used for pooling and the concentration and purity of DNA in the samples were controlled using a Nanodrop spectrophotometer, agarose gel electrophoresis and Qubit fluorometer. All participants in the scoliosis and preeclampsia studies have provided a written informed consent. The study protocols have been approved by the regional ethical board in Lund (290/2006), regional ethical board in Stockholm (2009/1124-31/2), research ethics committee of Lund University (LU 363-02), Karolinska Institutet (496/02), and Coordinating Ethics Committee of the Hospital District of Helsinki and Uusimaa (149/E0/07). The methods were carried out in accordance with the approved guidelines.

WGS: EDTA-blood samples (~3 mL) were collected from twenty privately owned Finnish Bull Terriers. DNA was extracted using the semi-automated Chemagen extraction robot (PerkinElmer Chemagen Technologie GmbH). Sample collections and study protocols were ethically approved by the Animal Ethics Committee of State Provincial Office of Southern Finland, Hämeenlinna, Finland (ESAVI/6054/04.10.03/ 2012). Twenty samples, 1 μg of each, were divided into two pools, 10 in each. One pool was composed of 10 Bull Terriers with compulsive tail-chasing behaviour^21^, while the other consisted of 10 controls. The concentration and purity of DNA in the samples were controlled using a Nanodrop spectrophotometer, agarose gel electrophoresis and Qubit fluorometer.

### Library preparation and NGS sequencing

Library preparation and whole exome sequencing on the pre-eclampsia study have been previously described^22^. The same setting was applied to the scoliosis study as well^17^. Because of the high PCR duplication rate in the first round among the 10 pooled scoliosis DNA samples, technical replications were performed for whole exome sequencing with the same library in eight pools. The DNA libraries for the whole genome sequencing were prepared using TruSeq DNA kits (Illumina Inc.) and the two pooled genomic DNA sample sets were fragmented to 300 bp. The clustering was performed on a cBot cluster generation system using a HiSeq paired-end read cluster generation kit (Illumina Inc.). The technical replication was performed for each pool with a different DNA library preparation. Both whole genome and exome sequencing were performed on an Illumina HiSeq 2000. The base conversion was done using OLB v1.9 (Illumina Inc.).

### NGS reads alignment

Initial quality control of the sequencing data was performed by the sequencing service provider (SciLifeLab Core Facility, Stockholm, Sweden). Paired-end Illumina sequence reads were aligned to the reference genomes with Burrows-Wheeler Aligner (BWA)^23^ version 0.6.1 for each pool. In the idiopathic scoliosis and pre-eclampsia studies, we used the National Center for Biotechnology Information (NCBI) human reference genome build 37 (GRCh37/hg19) as the reference genome. In the Bull Terrier study, the reference genome was *Canis lupus familiaris* genome assembly CanFam3.1. The threshold of the base quality score for read alignment was set to 20. SAMtools^24^ was used to remove PCR duplicates in each pool and evaluate read depths (version 0.1.18 for the WES studies and version 0.1.19 for the WGS study). Genome Analysis Toolkit (GATK)^25^ version 2.7.2 was applied for local realignment and base recalibration. The reference variant supplied for recalibration was dbSNP 137 in WES. In the WES studies, we used BEDtools^26^ version 2.16.2 for randomly selecting 70% and 80% reads in pre-eclampsia pool 7 to evaluate the influence of depth on variant detection and MAF estimation of the validated SNVs.

### SNV detection

We applied three tools, SAMtools mpileup function (version 0.1.19), GATK UnifiedGenotyper module (version 2.7.2 for the WES studies and version 3.2.2 for the WGS study), and Freebayes^27^ (version 0.9.21), to detect SNVs. All aligned reads from 10 pools in WES were simultaneously taken as input for variant detection tools. Uniquely mapped reads (mapping quality >20) with base quality score >20 were utilised for variant identification. The maximum read depth was set as 10,000x when using SAMtools and GATK. Besides the default diploid setting in SAMtools and GATK UnifiedGenotyper detection, we also applied a ploidy setting of 20 in GATK UnifiedGenotyper and Freebayes detections since each pool contained 10 individual samples in all three studies.

### Allele frequency estimation

Two strategies were applied for allele frequency estimation: based on read depth or allele count. SAMtools, GATK and Freebayes counted the read depth of reference and alternative alleles across all pooled samples on each SNV locus when detecting the variants. In the WES studies, the total AAF for validated SNVs in 100 samples was estimated by calculating the percentage of reads supporting the alternative allele across all 10 pools. Additionally, when using GATK and Freebayes with ploidy setting for variant detection, we used the ratio of alternative allele counts to total allele counts (n = 200) from genotype prediction in all pooled samples as total AAF. In the Bull Terrier study, the same strategy was applied for estimating MAF: the minor allele counts from genotype information generated by GATK and Freebayes using ploidy setting were extracted in each pooled samples, then divided by total alleles in the pool (n = 20).

### SNVs filtering for validation

In the WES studies, candidate SNVs potentially associated with the phenotypes were selected for genotyping validation. The following filtering criteria were applied to select candidate rare and low-frequency variants: 1. Filtering out singleton SNVs in only one case pool or SNVs in more than one control pools; 2. Keeping rare and low-frequency functional SNVs with read depth >10×; 3. Filtering out the SNVs located in ‘unreliable genes’ according to the suspect gene lists^28^,^29^. In the scoliosis study, we further kept the relevant SNVs according to the Gene Ontology Consortium database^30^. In addition, we also selected the SNVs that were not present in the control pools by manual visualization in Integrative Genomics Viewer, IGV^31^,^32^. In the downstream filtering of the pre-eclampsia study we also included some common variants present in more than one pre-eclampsia pool, but in less than five scoliosis pools. Taking the scoliosis data as an external reference together with the population-based reference from 1000 Genomes project^33^ and SiSu project (www.sisuproject.fi), the ratio of MAFs in all pre-eclampsia samples to MAFs in the reference dataset >1.5 was employed as the filtering threshold. In addition, one nonsense variant and one SNV located in a linkage peak region identified in the previous studies^19^ were selected.

### Genotyping

The SNV validation by genotyping has been previously described in the scoliosis study^17^ and the pre-eclampsia study^22^. In brief, the variants selected for validation were genotyped using Sequenom MassARRAY system (San Diego, California, United States) on the samples included in the WES. The 20 Bull Terriers included in the WGS were genotyped individually at FIMM Technology Centre under routine quality control by Illumina GenomeStudio (FIMM Technology Centre, University of Helsinki, Helsinki, Finland) using Illumina Canine HD 173k SNP array (San Diego, California, United States). PLINK software^34^ was used for analysing the genotyping data and for calculating MAFs.

### Evaluation of variant detection with different tools

In the WES studies, to evaluate the performance of different SNV detection tools, we utilised ANNOVAR^35^ package to annotate the detected SNVs with dbSNP 144 and the 1000 Genomes project (August 2015). The SNVs that were not annotated in dbSNP 144 were defined as potential novel SNVs. The allele frequencies of the samples with European ancestry in the 1000 Genomes project were used as a reference for categorising the SNVs. The SNVs with alternative allele frequencies (AAFs) less than 1% were classed as rare variants and those with AAFs between 1% and 5% were defined as low-frequency variants. The SNVs with AAFs over 5% were classified as common variants. SNVs without AF information in the 1000 Genomes project were defined as unknown. The shared SNVs were defined as polymorphisms with the same genotypes detected by at least two tools. The SNVs with more than one alternative allele were defined as multi-allelic SNVs.

In the Bull Terrier WGS study, to reduce the complexity of evaluating tool performance and estimated allele frequency, we filtered out the multi-allelic SNVs identified by any tool. We took the genotypes of 20 dogs from the Illumina array as true condition to evaluate the performance of the variant detection tools. The measurements used are defined as follows:

True positive (TP): the number of SNVs detected by both the WGS and the Illumina arrays; True negative (TN): the number of monomorphic loci that did not show SNVs in either the WGS or the Illumina array; False positive (FP): the number of SNVs detected by the WGS, but monomorphic in the Illumina array; False negative (FN): the number of SNVs detected by the Illumina array, but monomorphic in the WGS.


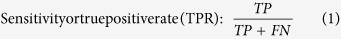



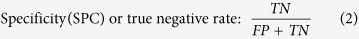



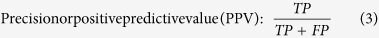



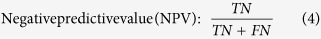



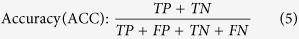


### Evaluation of MAF estimation

In the WES studies, we used root-mean-square deviation (RMSD) to measure the difference between MAF estimated from exome sequencing and experimentally validated MAF by genotyping. The RMSD were calculated as below:


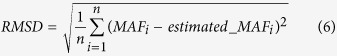


MAF: Experimentally validated minor allele frequency; estimated_MAF: minor allele frequency estimated from the exome sequencing data. In the Bull Terrier WGS study, we pooled minor allele counts of the 10 affected and 10 unaffected dogs according to the WGS setting and counted the minor alleles of each pooled sample in the array data. The allele count differences between the two platforms were calculated by directly comparing minor allele counts between the WGS and the Illumina arrays. Pearson correlation coefficient (*r*) was applied to measure the correlation between estimated MAF and experimentally validated MAF in all three studies.

## Additional Information

**How to cite this article**: Wang, J. *et al.* Investigation of rare and low-frequency variants using high-throughput sequencing with pooled DNA samples. *Sci. Rep.* **6**, 33256; doi: 10.1038/srep33256 (2016).

## Supplementary Material

Supplementary Information

## Figures and Tables

**Table 1:** Comparison of SNV detection by SAMtools, GATK, and Freebayes using multiple pools as simultaneous input.

Study		SAMtools	GATK (ploidy = 20)	Freebayes (ploidy = 20)
Idiopathic scoliosis	On enrichment regions&	79 677	171 286	219 526
	Annotated in dbSNP 144	74 098 (93.0%)	143 920 (84.0%)	134 575 (61.3%)
	Rare*	2 049 (2.6%)	20 414 (11.9%)	15 857 (7.2%)
	Low-frequency*	5 884 (7.4%)	24 790 (14.5%)	22 625 (10.3%)
	Common*	63 505 (79.7%)	71 560 (41.8%)	71 064 (32.4%)
	Unknown frequency	8 239 (10.3%)	54 522 (31.8%)	109 980 (50.1%)
Pre-eclampsia	On enrichment regions&	69 500	180 607	192 716
	Annotated in dbSNP 144	64 665 (93.0%)	153 740 (85.1%)	137 961 (71.6%)
	Rare*	583 (0.8%)	26 859 (14.9%)	20 066 (10.4%)
	Low-frequency*	2 329 (3.4%)	26 242 (14.5%)	23 576 (12.2%)
	Common*	59 320 (85.3%)	72 432 (40.1%)	71 984 (37.4%)
	Unknown frequency	7 268 (10.5%)	55 074 (30.5%)	77 090 (40.0%)
Bull Terrier	Total	4 736 038	7 323 018	7 612 527
	Mean depth >30×	4 253 121 (89.6%)	6 704 136 (91.5%)	6 756 976 (88.8%)
	On Illumina array	90 190	100 678	101 082

^&^The number of SNVs in the target regions captured by the Agilent SureSelect enrichment kit.

^*^Rare: alternative allele frequency <1%; Low-frequency: alternative allele frequency between 1% and 5%; Common: alternative allele frequency >5%. All of the allele frequencies were retrieved from the 1000 Genome European population (August 2015).

**Table 2:** Evaluation of variant detection tools in the Bull Terrier study.

Tool	Sensitivity (%)	Specificity (%)	Precision (%)	Negative Predictive Value (%)	Accuracy (%)
SAMtools	91.04	88.09	79.74	95.02	89.09
GATK (ploidy = 20)	96.59	97.62	96.24	97.85	97.22
Freebayes (ploidy = 20)	96.79	97.73	96.42	97.97	97.37
