# Supplementary material for: Investigation of rare and low-frequency variants using high-throughput sequencing with pooled DNA samples
Source: Sci Rep. 2016 Sep 16;6:33256. doi: 10.1038/srep33256 (PMC5025741; doi:10.1038/srep33256)

Investigation of rare and low-frequency variants using high-throughput sequencing with pooled DNA samples

Jingwen Wang[1,2], Tiina Skoog[1], Elisabet Einarsdottir[1,3], Tea Kaartokallio[4], Hannele Laivuori[4,5,6], Anna Grauers[7,8], Paul Gerdhem[7], Marjo Hytönen[9], Hannes Lohi[9], Juha Kere[1,2,3], Hong Jiao*[1,2]

[1]Department of Biosciences and Nutrition, Karolinska Institutet, *SE-14183* Huddinge, Sweden

[2]Science for Life Laboratory, Stockholm, Sweden

[3]Molecular Neurology Research Program, University of Helsinki and Folkhälsan Institute of Genetics, Helsinki, Finland

[4]Medical and Clinical Genetics, University of Helsinki and Helsinki University Hospital, Helsinki, Finland

[5]Obstetrics and Gynecology, University of Helsinki and Helsinki University Hospital, Helsinki, Finland

[6]Institute for Molecular Medicine Finland, University of Helsinki, Helsinki, Finland

[7]Department of Orthopedics, Karolinska University Hospital and Department of Clinical Sciences, Intervention and Technology (CLINTEC) Karolinska Institutet, Stockholm, Sweden

[8]Department of Orthopaedics, Sundsvall and Harnosand County Hospital, Sundsvall, Sweden

[9]Department of Veterinary Biosciences, and Research Programs Unit, Molecular Neurology, University of Helsinki and Folkhälsan Research Center, Helsinki, Finland

*Corresponding author, hong.jiao@ki.se

Table S1. Sequencing read alignment

		#Total reads	#Mapped reads	Mapped reads %	#Mapped reads after PCR duplicates removal	PCR duplicates rate
Idiopathic Scoliosis pools	IS_pool1	350 818 724	345 328 942	98.44%	162 639 797	53.64%
	IS_pool2	318 770 312	313 097 616	98.22%	160 274 578	49.72%
	IS_pool3	411 814 190	384 178 245	93.29%	249 296 807	39.46%
	IS_pool4	338 059 520	331 500 257	98.06%	149 723 410	55.71%
	IS_pool5	307 016 632	302 280 460	98.46%	160 789 962	47.63%
	IS_pool6	362 993 486	357 117 766	98.38%	175 026 048	51.78%
	IS_pool7	312 455 272	306 657 667	98.14%	128 019 028	59.03%
	IS_pool8	290 847 924	286 088 829	98.36%	109 866 945	62.23%
	IS_pool9	313 332 730	306 391 993	97.78%	152 742 224	51.25%
	IS_pool10	346 329 114	340 341 111	98.27%	174 233 065	49.69%
Pre-eclampsia pools	PE_pool1	487 712 078	482 231 326	98.88%	80 204 504	83.55%
	PE_pool2	477 577 208	473 748 914	99.20%	196 121 726	58.93%
	PE_pool3	473 110 952	469 467 831	99.23%	245 303 433	48.15%
	PE_pool4	343 764 436	340 509 711	99.05%	224 338 206	34.74%
	PE_pool5	311 980 504	309 134 264	99.09%	200 585 344	35.71%
	PE_pool6	337 009 602	332 842 828	98.76%	224 518 107	33.38%
	PE_pool7	290 844 422	288 530 656	99.20%	188 971 302	35.03%
	PE_pool8	450 485 536	447 265 933	99.29%	240 281 714	46.66%
	PE_pool9	311 220 698	309 026 352	99.29%	221 173 298	28.93%
	PE_pool10	445 419 316	441 329 378	99.08%	141 853 221	68.15%
Bull Terriers pools	Bull_pool1	3 425 241 184	3 291 960 157	96.11%	3 201 059 572	6.54%
	Bull_pool2	3 861 839 148	3 508 056 427	90.84%	3 198 338 582	17.18%

Table S2. Read depth and coverage in each pooled sample

		>=60x	>=30x	0x	mean depth
Idiopathic Scoliosis pools*	IS_pool1	74.63%	87.42%	0.76%	155.22
	IS_pool2	79.24%	88.87%	0.74%	194.48
	IS_pool3	74.18%	86.56%	1.03%	163.55
	IS_pool4	78.35%	88.64%	0.74%	178.99
	IS_pool5	76.98%	88.12%	0.79%	170.42
	IS_pool6	72.04%	85.60%	1.13%	155.10
	IS_pool7	62.51%	81.41%	1.32%	114.62
	IS_pool8	55.87%	77.24%	2.03%	104.35
	IS_pool9	71.73%	86.00%	0.99%	147.21
	IS_pool10	74.15%	86.64%	1.02%	163.92
	All pools	96.13%	97.77%	0.29%	
Pre-eclampsia pools*	PE_pool1	52.67%	76.00%	0.88%	95.32
	PE_pool2	83.66%	91.22%	0.69%	221.49
	PE_pool3	85.58%	91.54%	0.77%	277.60
	PE_pool4	83.90%	91.03%	0.72%	242.90
	PE_pool5	82.78%	90.60%	0.73%	219.88
	PE_pool6	83.31%	90.63%	0.75%	244.67
	PE_pool7	82.41%	90.49%	0.73%	207.93
	PE_pool8	86.64%	92.61%	0.64%	267.03
	PE_pool9	86.16%	92.27%	0.68%	251.18
	PE_pool10	73.28%	89.67%	0.63%	169.46
	All pools	97.89%	98.82%	0.51%	
Bull Terriers pools	Bull_pool1	92.71%	97.38%	0.93%	132.98
	Bull_pool2	90.82%	97.06%	0.96%	135.28

* read depth and coverage were measured based on Agilent SureSelect enrichment kit target regions.

Table S3. SNVs identified by three variant detection tools in the WES studies

		SAMTools		GATK (ploidy = 20)		Freebayes (ploidy = 20)	
		Amount	Percentage (%)	Amount	Percentage (%)	Amount	Percentage (%)
Idiopathic scoliosis	Total	2 115 514		2 206 799		2 059 383	
	Mean depth ≥ 10x	193 796	9.16	376 132	17.04	504 111	24.48
	Singleton	486 385	22.99	511 978	23.20	926 843	45.01
	On enrichement regions &	79 677	3.77	171 286	7.76	219 526	10.66
	Annotated in dbSNP	1 927 069	91.09	1 923 112	87.14	1 479 739	71.85
	Rare*	15 230	0.79	44 182	2.30	44 975	3.04
	Low-frequency*	45 217	2.14	80 852	3.66	80 972	3.93
	Common*	1 775 611	83.93	1 632 163	73.96	1 213 635	58.93
	Unknown frequency	279 457	13.21	449 603	20.37	719 801	34.95
Preeclampsia	Total	2 841 127		3 169 180		2 385 543	
	Mean depth ≥ 10x	242 913	8.55	531 373	16.77	595 190	24.95
	Singleton	469 717	16.53	472 612	14.91	770 238	32.29
	On enrichement regions &	69 500	2.45	180 607	5.70	192 716	8.08
	Annotated in dbSNP	2 601 581	91.57	2 787 601	87.96	1 823 259	76.43
	Rare	15 747	0.55	71 563	2.26	67 401	2.83
	Low-frequency	48 954	1.72	113 517	3.58	102 934	4.31
	Common	2 424 753	85.34	2 378 168	75.04	1 489 652	62.44
	Unknown frequency	351 674	12.38	605 932	19.12	725 556	30.41

& The number of SNVs in the target regions captured by the Agilent SureSelect enrichment kit

*Rare: alternative allele frequency < 1%; Low-frequency: alternative allele frequency between 1% and 5%; Common: alternative allele frequency > 5%.

All of the allele frequencies were retrieved from the 1000 Genome European population (August 2015).

Table S4. SNVs selected for genotyping validation

Project	Chromosome	Position	Ref	Alt	Function	Gene	Exonic function	dbSNP 144	MAF in 1K Genomes	Comments
	1	2435738	G	A	exonic	PLCH2	nonsynonymous SNV	rs114371513	0.0229	Failed for genotyping
	1	2436303	A	C	exonic	PLCH2	nonsynonymous SNV	rs79405490	0.0229	Failed for genotyping
	1	33160644	T	G	exonic	SYNC	nonsynonymous SNV	rs3795424	0.0298	
	1	33836164	G	A	exonic	PHC2	nonsynonymous SNV	rs41265897	0.0358	
	1	43651024	C	G	exonic	CFAP57	nonsynonymous SNV	rs74857529	0.0089	
	1	85742023	C	A	exonic	BCL10	nonsynonymous SNV	rs12037217	0.0268	
	1	110231746	A	G	exonic	GSTM1	nonsynonymous SNV	rs147668562	0.0288	Failed for primer design
	1	179312753	G	A	exonic	SOAT1	nonsynonymous SNV	rs141826063	0.001	
	1	181018212	G	A	exonic	MR1	nonsynonymous SNV	rs41268456	0.0109	Failed for primer design
	1	208252715	A	C	exonic	PLXNA2	nonsynonymous SNV	rs202222167		Monomorphic
	2	42281312	G	A	exonic	PKDCC	nonsynonymous SNV	rs34372645	0.0099	
	2	220284876	C	T	exonic	DES	nonsynonymous SNV	rs41272699	0.0189	
	2	242312572	G	A	exonic	FARP2	nonsynonymous SNV	rs61739702	0.0348	
	3	52394055	G	A	exonic	DNAH1	nonsynonymous SNV	rs61734638	0.0239	
	5	140736814	G	C	exonic	PCDHGA4	nonsynonymous SNV	rs11575951	0.0258	
	5	140754392	C	A	exonic	PCDHGA6	nonsynonymous SNV	rs11575953	0.0249	
	6	31935750	G	A	exonic	SKIV2L	nonsynonymous SNV		0.0338	
	6	43469299	G	A	exonic	TJAP1	nonsynonymous SNV	rs140868873	0.0089	Failed for genotyping
	6	151270231	G	A	exonic	MTHFD1L	nonsynonymous SNV	rs61748674	0.0119	
	7	43921261	T	C	exonic	URGCP,URGC	nonsynonymous SNV	rs41279597	0.0258	
	7	44747578	G	A	exonic	OGDH	nonsynonymous SNV	rs2070607	0.0417	
	7	100465824	G	A	exonic	TRIP6	nonsynonymous SNV	rs2437100	0.0268	
	7	123593764	T	C	exonic	SPAM1	nonsynonymous SNV	rs34633019	0.0199	
	9	34660864	C	T	exonic	IL11RA	nonsynonymous SNV	rs11575580	0.0239	
Idiopathic	10	86131671	A	G	exonic	CCSER2	nonsynonymous SNV	rs150621117	0.007	
Scoliosis	10	97143826	T	C	exonic	SORBS1	nonsynonymous SNV	rs35808802	0.0537	
	11	71725634	C	T	exonic	NUMA1	nonsynonymous SNV	rs149184541	0.0109	
	11	124742385	G	A	exonic	ROBO3	nonsynonymous SNV	rs4935898	0.0348	
	11	126343227	T	C	exonic	KIRREL3	nonsynonymous SNV	rs111418068	0.0447	
	11	134128923	C	G	exonic	ACAD8	nonsynonymous SNV	rs113488591	0.0199	
	12	7355269	T	C	exonic	PEX5	nonsynonymous SNV	rs76708142	0.001	
	12	15656846	C	G	exonic	PTPRO	nonsynonymous SNV	rs61754411	0.0229	
	12	49176805	C	T	exonic	ADCY6	nonsynonymous SNV	rs115315671	0.0239	
	12	55420891	A	G	exonic	NEUROD4	nonsynonymous SNV	rs118105704	0.0189	
	12	56092668	C	T	exonic	ITGA7	nonsynonymous SNV	rs74867235	0.0427	Failed for primer design
	15	42434824	C	T	exonic	PLA2G4F	nonsynonymous SNV	rs28674123	0.0278	
	15	99762041	C	T	exonic	TTC23	nonsynonymous SNV	rs78534478	0.0358	
	15	101109904	T	C	exonic	LINS1	nonsynonymous SNV	rs141855950	0.006	
	16	15661871	A	G	exonic	C16orf45	nonsynonymous SNV	rs149928639	0.001	
	17	7189048	G	A	exonic	SLC2A4	nonsynonymous SNV	rs121434581	0.007	
	17	44060859	A	G	exonic	MAPT	nonsynonymous SNV	rs63750072	0.0477	
	19	1236080	A	G	exonic	CBARP	nonsynonymous SNV	rs113298601	0.0169	Failed for primer design
	19	18897440	T	C	exonic	COMP	nonsynonymous SNV	rs61739916	0.0487	
	19	19790178	A	G	exonic	ZNF101	nonsynonymous SNV	rs59327775	0.0268	
	19	45296806	C	T	exonic	CBLC	nonsynonymous SNV	rs3208856	0.0229	
	21	47404302	G	A	exonic	COL6A1	nonsynonymous SNV	rs11553519	0.0527	
	21	47703705	T	C	exonic	MCM3AP	nonsynonymous SNV	rs144151494	0.002	
	22	38328597	A	G	exonic	MICALL1	nonsynonymous SNV	rs34834842	0.0298	
	22	46657308	T	C	exonic	PKDREJ	nonsynonymous SNV	rs41302599	0.0199	
	X	31986607	G	A	exonic	DMD	nonsynonymous SNV	rs1800273	0.0326	
	1	16133987	T	C	exonic	UQCRHL	nonsynonymous SNV	rs7417535	0.0994	
	1	17271989	G	A	exonic	CROCC	nonsynonymous SNV	rs147139590		Failed for primer design
	1	165619079	C	T	UTR5	MGST3		rs6681	0.0497	
	1	165648710	G	A	exonic	ALDH9A1	nonsynonymous SNV	rs1143660	0.0318	
	1	240371426	T	C	exonic	FMN2	nonsynonymous SNV	rs200640213	0.0974	Failed for primer design
	2	42991200	C	G	exonic	OXER1	nonsynonymous SNV	rs34142793	0.0229	
	2	113999154	A	G	exonic	PAX8	nonsynonymous SNV	rs201436651		Failed for primer design
	2	127451514	G	A	exonic	GYPC	nonsynonymous SNV	rs111631066	0.0229	Failed for primer design
	2	178879181	G	A	exonic	PDE11A	stopgain	rs76308115	0.005	Failed for primer design
	3	43591327	G	A	exonic	ANO10	nonsynonymous SNV	rs17409162	0.0487	
	4	843782	G	T	exonic	GAK	nonsynonymous SNV	rs138216547	0.002	
	4	983612	C	T	exonic	SLC26A1	nonsynonymous SNV	rs73219719	0.0089	
	4	9250422	T	C	exonic	USP17L11,US	nonsynonymous SNV	rs376110430	0.3171	Failed for primer design
	4	42020142	A	G	exonic	SLC30A9	nonsynonymous SNV	rs2581423	0.0268	
	6	170058374	C	T	exonic	WDR27	nonsynonymous SNV	rs3800544	0.0716	
	7	127233977	G	T	exonic	FSCN3	nonsynonymous SNV	rs3779536	0.0258	Failed for primer design
	7	150746352	G	A	exonic	ASIC3	nonsynonymous SNV	rs145775749	0.004	
	8	2021547	G	A	exonic	MYOM2	nonsynonymous SNV		0.0219	
	9	6421105	G	A	exonic	UHRF2	nonsynonymous SNV	rs140727862	0.001	
	9	130263351	G	A	exonic	LRSAM1	nonsynonymous SNV	rs140786088	0.007	
	9	136915695	G	A	exonic	BRD3	nonsynonymous SNV	rs34609592	0.0129	
	10	50018765	G	A	exonic	WDFY4	nonsynonymous SNV	rs61742644	0.0437	
	10	135051653	A	G	exonic	VENTX	nonsynonymous SNV	rs2240891	0.008	Failed for primer design
	11	430339	C	G	exonic	ANO9	nonsynonymous SNV	rs113926353	0.0606	
	11	17538989	C	T	exonic	USH1C	nonsynonymous SNV	rs116996553		
	11	35327767	A	T	exonic	SLC1A2	nonsynonymous SNV	rs180817412	0.002	
	11	46430081	C	A	exonic	AMBRA1	nonsynonymous SNV	rs72910100	0.0189	
	11	76813961	C	T	exonic	OMP	nonsynonymous SNV		0.0517	
	11	134182375	G	A	exonic	GLB1L3	nonsynonymous SNV	rs2509062	0.003	
Preeclampsia	12	7548969	C	A	exonic	CD163L1	nonsynonymous SNV	rs145411783	0.007	
	12	29786126	G	A	exonic	TMTC1	nonsynonymous SNV	rs142394560	0.0129	
	12	57397033	C	T	exonic	ZBTB39	nonsynonymous SNV	rs61752546	0.0258	
	12	101750814	T	A	exonic	UTP20	nonsynonymous SNV	rs10082778	0.0139	
	12	105521009	C	A	exonic	KIAA1033	nonsynonymous SNV	rs199570381		Monomorphic
	13	21563311	G	A	exonic	LATS2	nonsynonymous SNV	rs77919685	0.0249	
	14	33014869	C	T	exonic	AKAP6	nonsynonymous SNV	rs3742926	0.1004	
	15	31776837	C	G	exonic	OTUD7A	nonsynonymous SNV	rs76704217	0.0378	
	15	43724532	T	C	exonic	TP53BP1	nonsynonymous SNV	rs3803339	0.005	
	15	50555544	G	A	exonic	HDC	nonsynonymous SNV	rs17740607	0.0974	
	15	53081859	G	C	exonic	ONECUT1	nonsynonymous SNV	rs74805019	0.0378	Failed for primer design
	15	75651981	G	A	exonic	MAN2C1	nonsynonymous SNV	rs78610801	0.0328	
	16	23160057	C	T	exonic	USP31	nonsynonymous SNV	rs139702277	0.0129	tagged with SNP rs117741116
	17	3635740	C	G	exonic	ITGAE	nonsynonymous SNV	rs3744679	0.0497	
	17	14110451	A	C	exonic	COX10	nonsynonymous SNV	rs200435051		Monomorphic
	17	74878259	G	A	exonic	MGAT5B	nonsynonymous SNV	rs571264	0.1431	
	18	53303150	T	C	UTR5	TCF4		rs77891683	0.0219	
	19	3834863	C	T	exonic	ZFR2	nonsynonymous SNV	rs61747120	0.0408	
	19	5827765	G	A	exonic	NRTN	nonsynonymous SNV	rs79744308	0.0417	
	19	10224548	G	A	exonic	P2RY11,PPAN	nonsynonymous SNV	rs3745601	0.1083	tagged with SNP rs12462506

19	11508177	G	A	exonic	RGL3	nonsynonymous SNV	rs2291516	0.0915	
19	43990795	G	A	exonic	PHLDB3	nonsynonymous SNV	rs117243018	0.0308	
20	20033223	A	T	exonic	CRNKL1	nonsynonymous SNV	rs2273056	0.0586	
20	32664601	C	A	exonic	RALY	nonsynonymous SNV	rs148075348	0.0099	
20	43384935	C	G	exonic	RIMS4	nonsynonymous SNV	rs199569233		Monomorphic
22	19950263	G	T	exonic	COMT	nonsynonymous SNV	rs6267	0.007	Failed for primer design
X	2724760	T	C	exonic	XG	nonsynonymous SNV	rs3749988	0.0091	Failed for primer design
X	138897130	A	C	exonic	ATP11C	nonsynonymous SNV	rs2491014	0.03	
X	140967121	A	T	exonic	MAGEC3	nonsynonymous SNV	rs12852593	0.0196	
X	153594535	G	A	exonic	FLNA	nonsynonymous SNV		0.0157	

Table S5. Root-mean-squared deviation (RMSD) of MAF estimation by different tools

		Scoliosis	Preeclampsia	Total
Calculated based on read depths *	SAMtools	0.028	0.039	0.034
	GATK	0.045	0.066	0.057
	Freebayes	0.023	0.031	0.028
Calculated based on allele counts #	GATK	0.031	0.033	0.032
	Freebayes	0.029	0.032	0.031

*MAF = minor allele read depths / total read depths

#MAF = minor allele counts predicted by software / total allele counts

Table S6. SNVs detection comparison between the whole genome sequencing data and the Illumina array data

SAMTools		Whole genome sequencing		
Affected		Non-Monomorphic	Monomorphic	Total
Illumina SNP array	Non-Monomorphic	51481	13091	64572
	Monomorphic	5747	99968	105715
	Total	57228	113059	170287

SAMTools		Whole genome sequencing		
Unaffected		Non-Monomorphic	Monomorphic	Total
Illumina SNP array	Non-Monomorphic	53914	13681	67595
	Monomorphic	4630	98035	102665
	Total	67595	102665	170260

SAMTools		Whole genome sequencing		
Total		Non-Monomorphic	Monomorphic	Total
Illumina SNP array	Non-Monomorphic	105395	26772	132167
	Monomorphic	10377	198003	208380
	Total	115772	224775	340547

GATK		Whole genome sequencing		
Affected		Non-Monomorphic	Monomorphic	Total
Illumina SNP array	Non-Monomorphic	62374	2198	64572
	Monomorphic	2195	103520	105715
	Total	64572	105715	170287

GATK		Whole genome sequencing		
Unaffected		Non-Monomorphic	Monomorphic	Total
Illumina SNP array	Non-Monomorphic	64827	2768	67595
	Monomorphic	2292	100373	102665
	Total	67595	102665	170260

GATK		Whole genome sequencing		
Total		Non-Monomorphic	Monomorphic	Total
Illumina SNP array	Non-Monomorphic	127201	4966	132167
	Monomorphic	4487	203893	208380
	Total	131688	208859	340547

Freebayes		Whole genome sequencing		
Affected		Non-monomorphic	Monomorphic	Total
Illumina SNP array	Non-monomorphic	62381	2191	64572
	Monomorphic	2074	103641	105715
	Total	64455	105832	170287

Freebayes		Whole genome sequencing		
Unaffected		Non-Monomorphic	Monomorphic	Total
Illumina SNP array	Non-Monomorphic	65054	2541	67595
	Monomorphic	2153	100512	102665
	Total	67207	103053	170260

Freebayes		Whole genome sequencing		
Total		Non-Monomorphic	Monomorphic	Total
Illumina SNP array	Non-Monomorphic	127435	4732	132167
	Monomorphic	4227	204153	208380
	Total	131662	208885	340547

Table S7. Total number of SNVs identified with GATK in the genome and the enrichment regions of each pool

	Average Depth §	Variants in whole genome		Variants in the enrichment regions	
		Amount	Percentage*	Amount	Percentage #
IS_pool1	155.22	1 107 306	50.18%	169 274	99.64%
IS_pool2	194.48	1 337 422	60.60%	169 213	99.61%
IS_pool3	163.55	1 049 642	47.56%	169 013	99.49%
IS_pool4	178.99	1 216 604	55.13%	169 224	99.62%
IS_pool5	170.42	1 175 684	53.28%	169 227	99.62%
IS_pool6	155.10	1 085 013	49.17%	168 827	99.38%
IS_pool7	114.62	961 750	43.58%	168 572	99.23%
IS_pool8	104.35	864 769	39.19%	167 757	98.75%
IS_pool9	147.21	1 074 687	48.70%	168 961	99.46%
IS_pool10	163.92	1 156 685	52.41%	168 881	99.41%
PE_pool1	95.32	964 967	30.45%	178 165	99.36%
PE_pool2	221.49	1 758 406	55.48%	178 793	99.71%
PE_pool3	277.60	1 981 116	62.51%	178 710	99.67%
PE_pool4	242.90	1 887 181	59.55%	178 699	99.66%
PE_pool5	219.88	1 797 343	56.71%	178 649	99.63%
PE_pool6	244.67	1 840 029	58.06%	178 651	99.64%
PE_pool7	207.93	1 750 191	55.23%	178 652	99.64%
PE_pool8	267.03	1 943 001	61.31%	178 855	99.75%
PE_pool9	251.18	1 981 906	62.54%	178 787	99.71%
PE_pool10	169.46	1 350 675	42.62%	178 767	99.70%
Bull_pool1	132.98	7 311 939	99.85%		
Bull_pool2	135.28	7 308 747	99.80%		

§ Average depth in the enrichment regions

*Column D represent the ratio of SNVs of each pool to all SNVs in the genome

Column F represents the ratio of SNVs of each pool to all SNVs in the enrichment regions

Figure S1. Workflow of pooled DNA sequencing processes

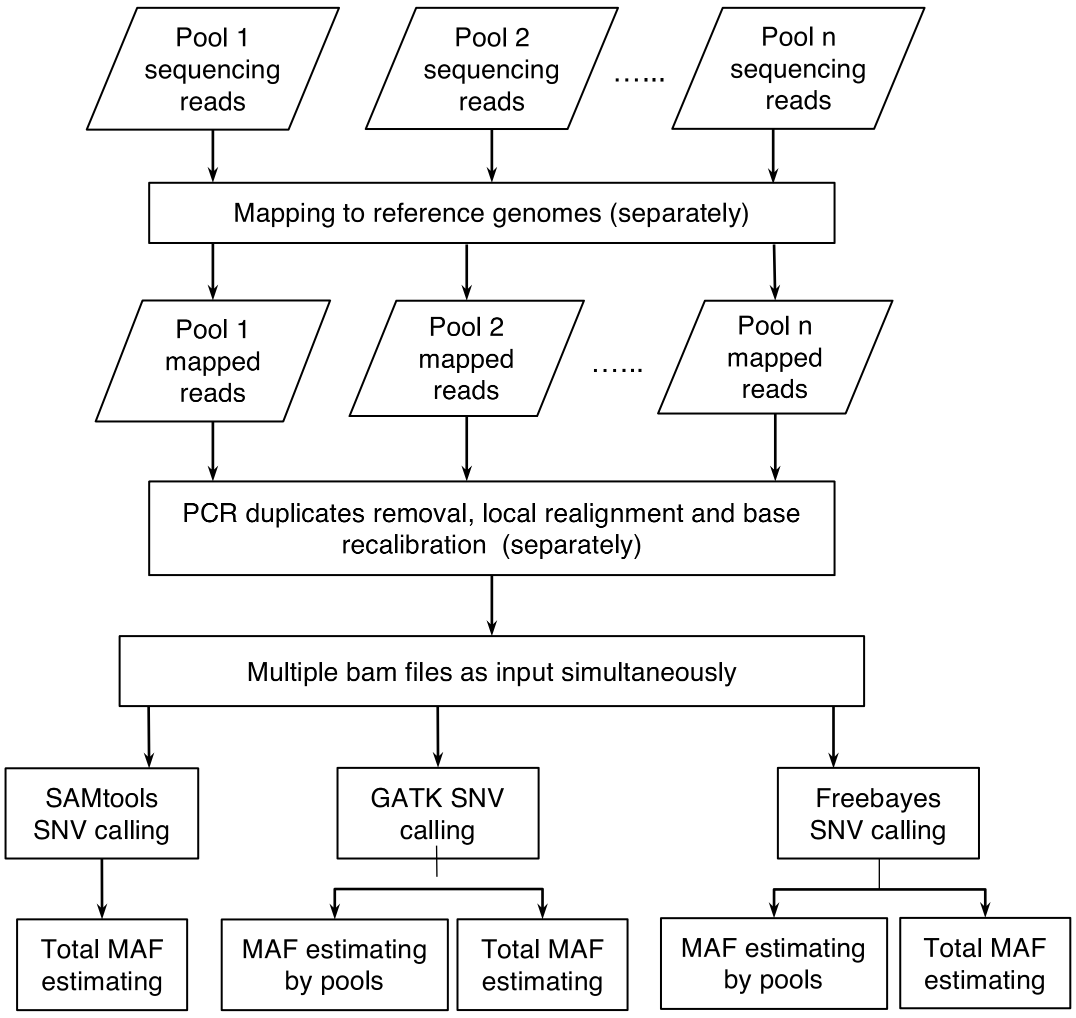

Figure S2. Agreement of the detected SNVs between using GATK diploid setting and ploidy of 20 setting
a) idiopathic scoliosis study, b) preeclampsia study

a

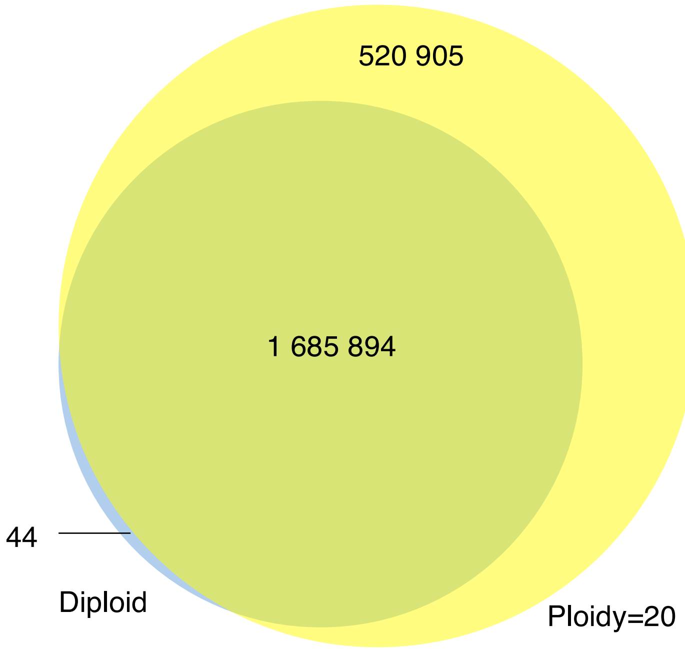

b

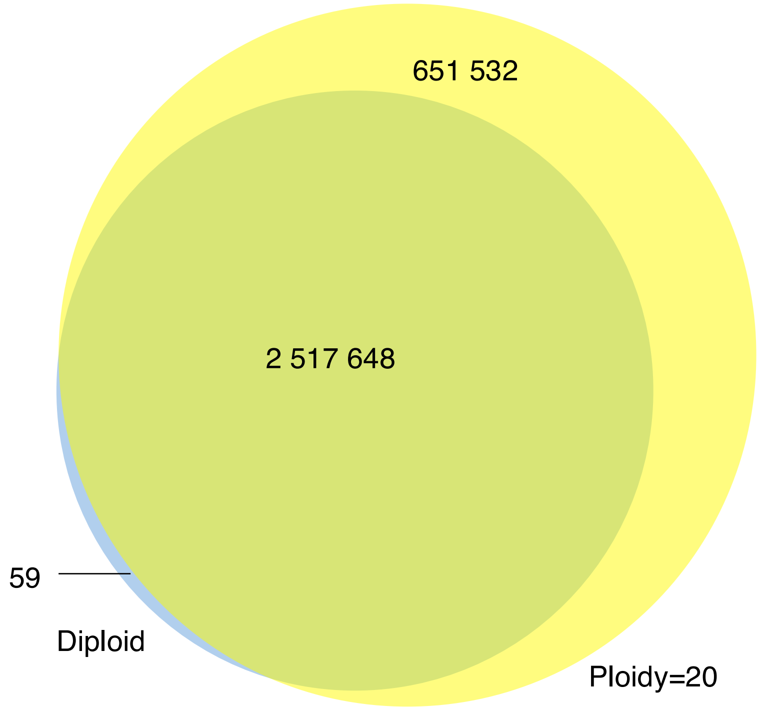

Figure S3. SNVs identified in different numbers of pools in the enrichment regions and the distribution of read depth at singleton SNV loci.
Mean depth represents the average depth of uniquely mapped reads at SNV loci.

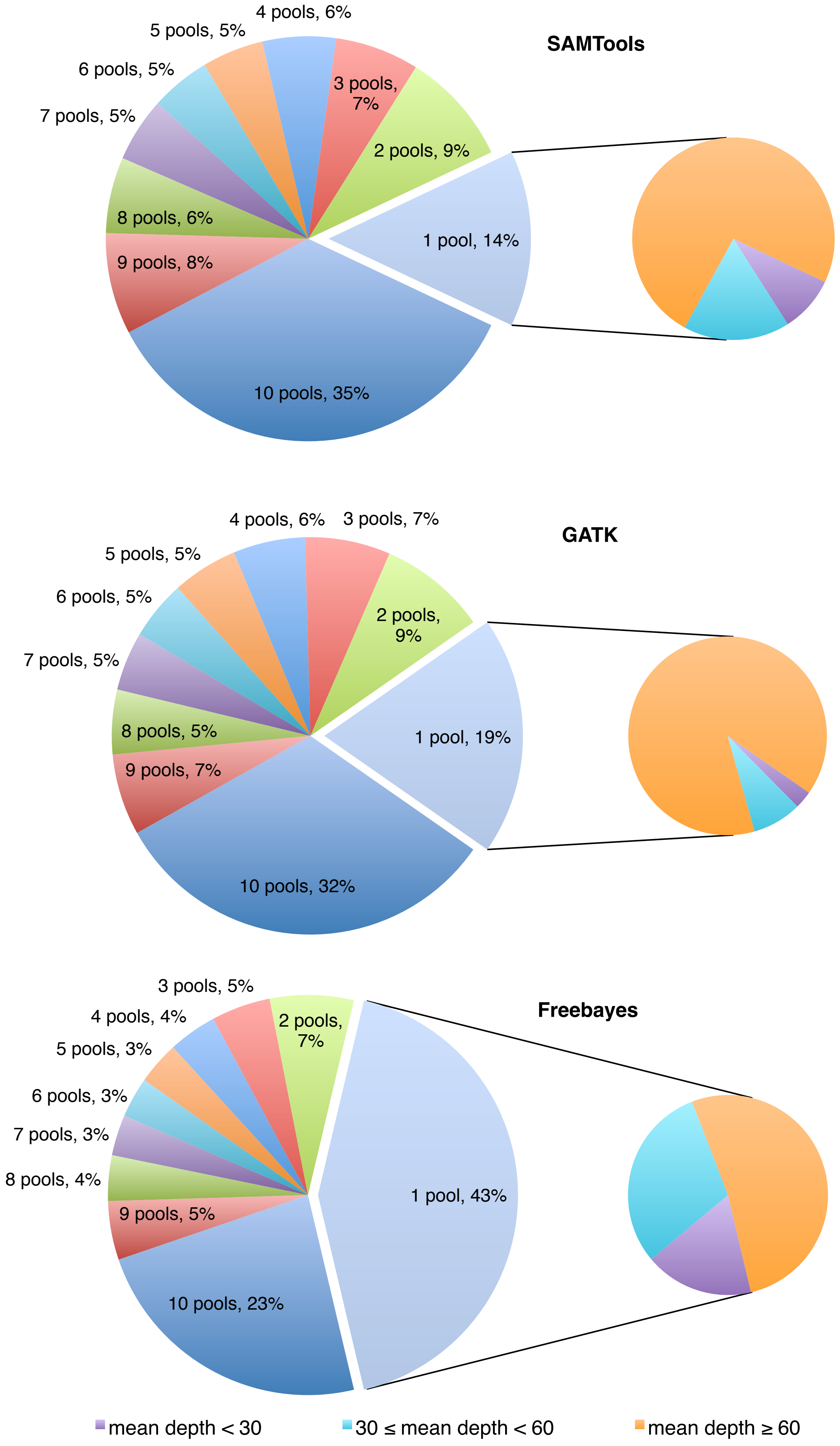

Figure S4. Distribution of SNVs uniquely detected by Freebayes

a) Mean depth among 10 pools, b) Numbers of pools where each SNV presented, c) Alternative allele frequency (AAF) in 1000 Genomes project database (rare: AAF<1%; low-frequency: 1%≤AAF≤5%; common: AAF>5%), d) Variant detection quality (SNV quality)

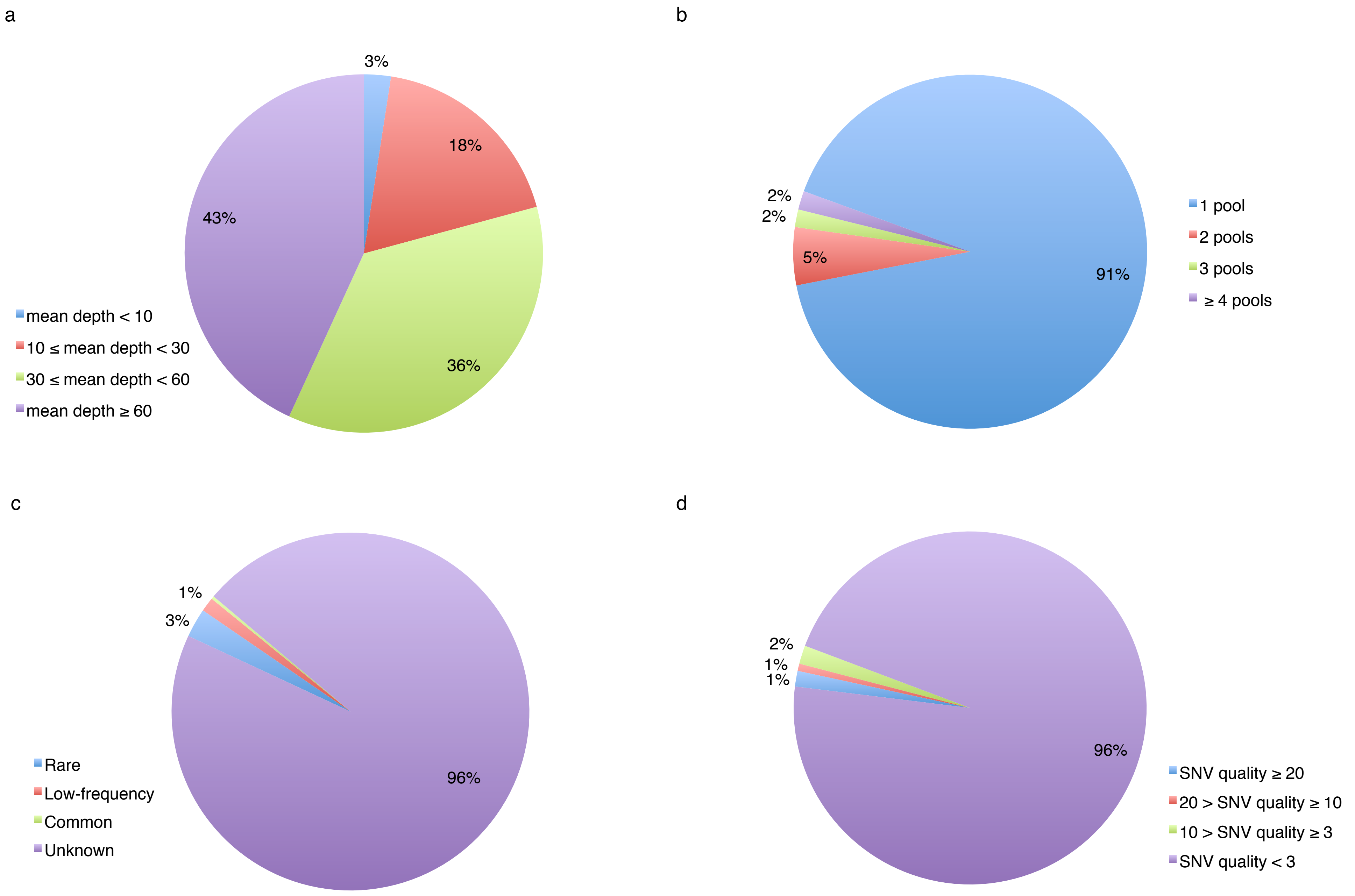

Figure S5. Comparison between the estimated MAF and the experimentally validated MAF
The MAFs were calculated based on read depths. The diagonal is shown with the grey dashed line. a) Idiopathic scoliosis, b) Pre-eclampsia

a

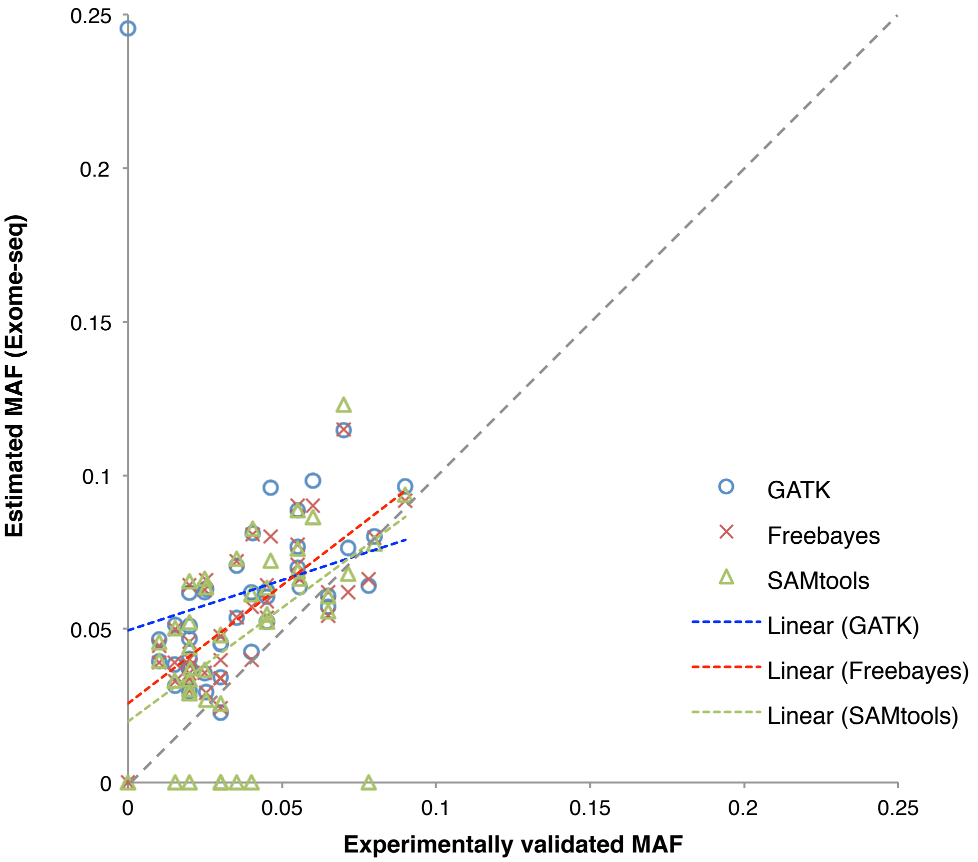

b

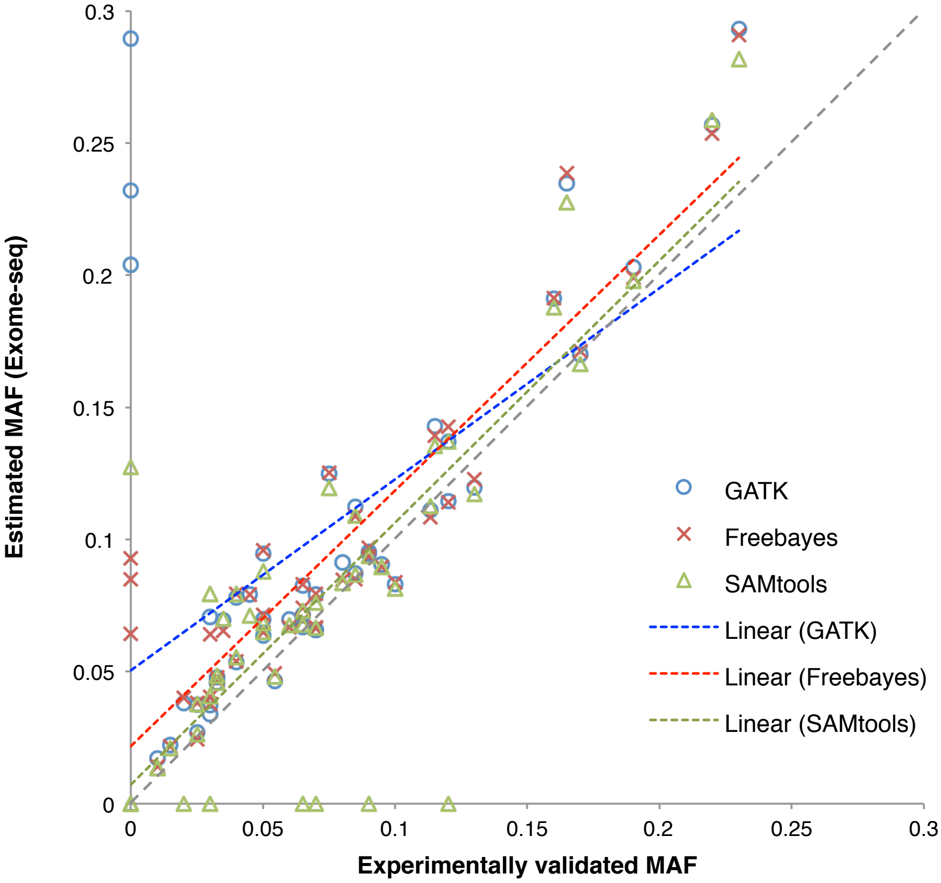

Figure S6. The accuracy of MAF estimated with GATK (ploidy setting) based on allele counts

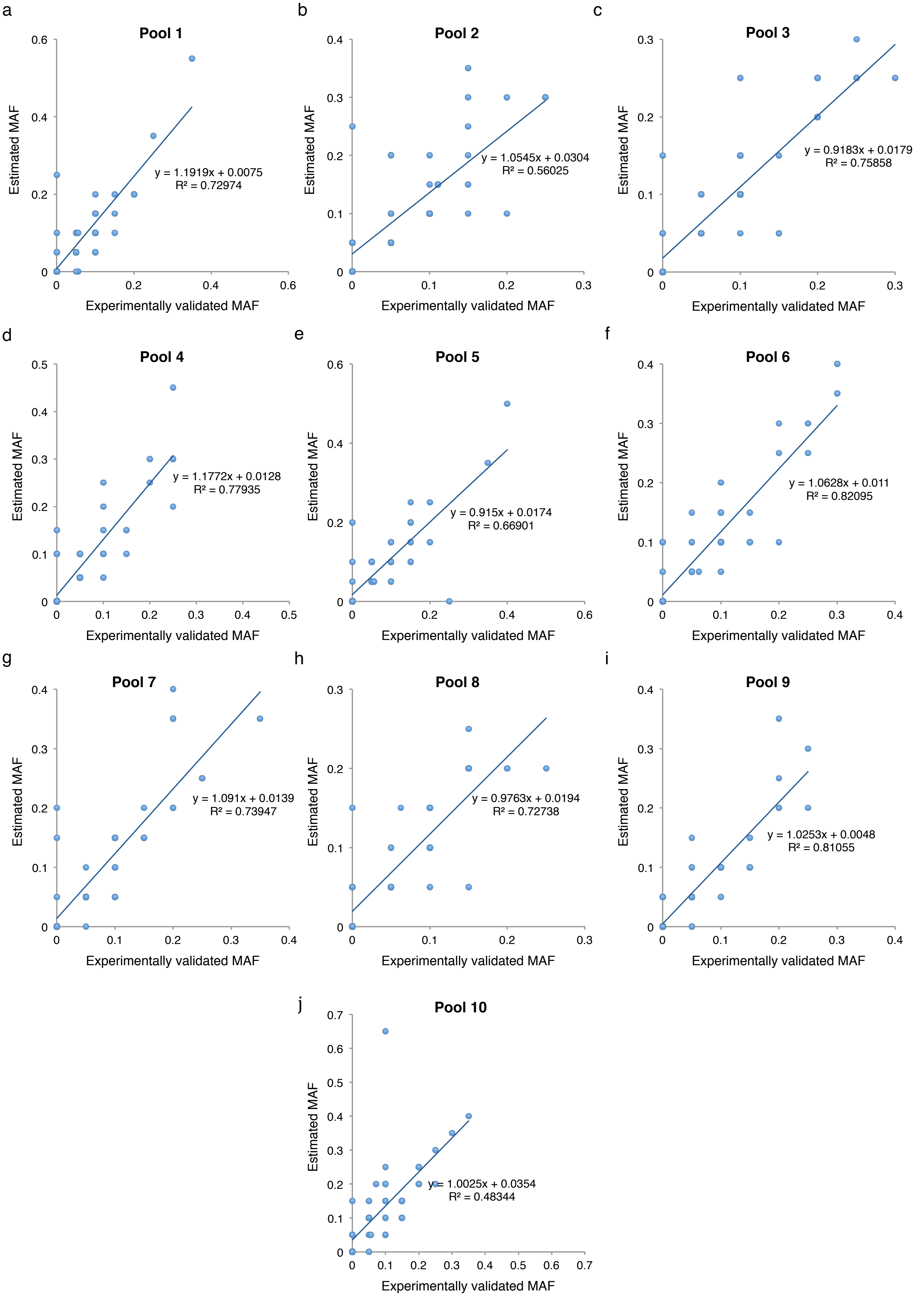

Figure S7. The SNVs with overestimated MAFs in the pre-eclampsia study
The MAFs were estimated with GATK based on allele counts in each pool. a) rs79744308, b) rs2581423, c) rs12852593, d) rs73219719

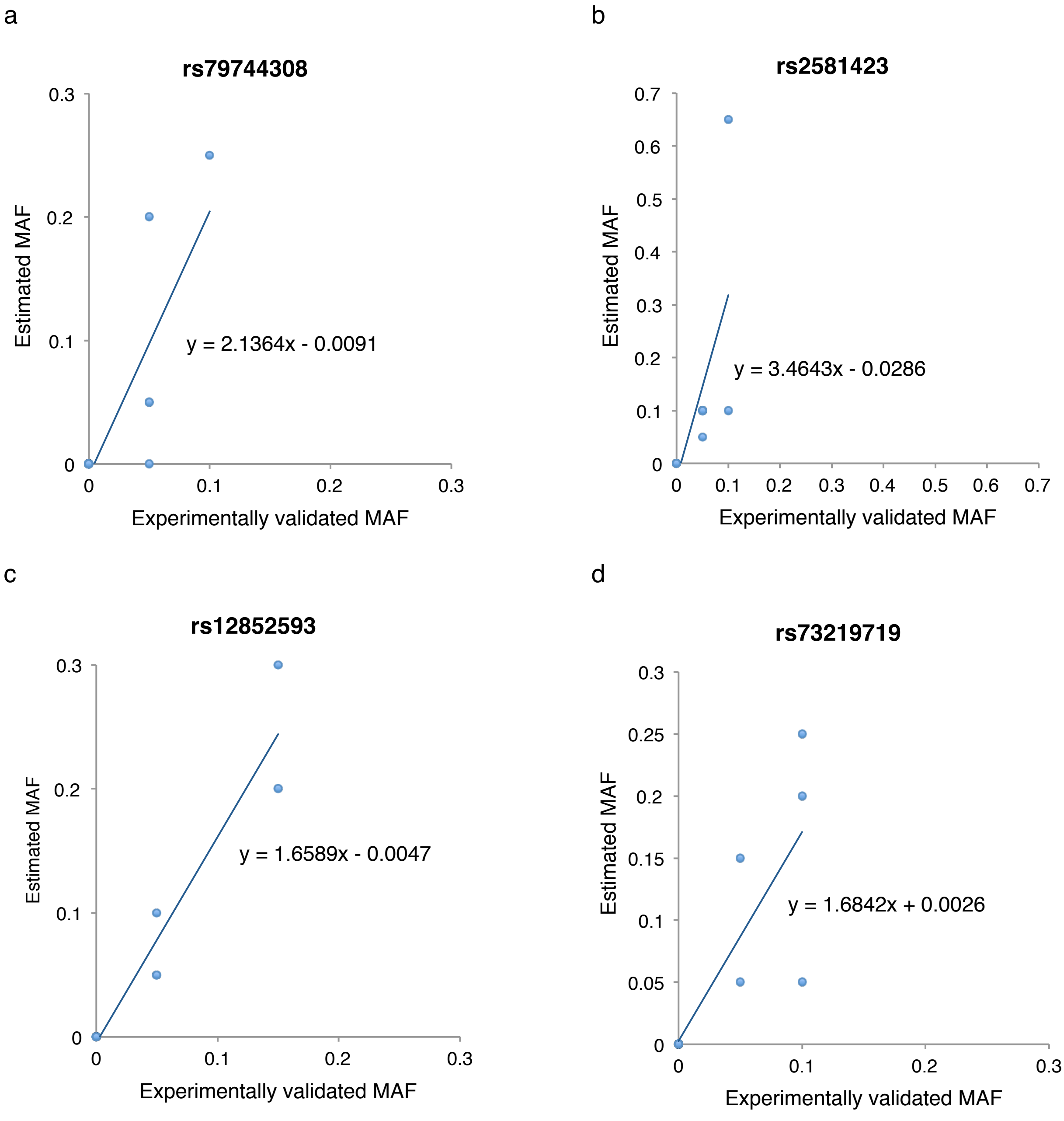

Figure S8. Differentiation between validated MAF and estimated MAF in the pre-eclampsia pool 7 by using 70%, 80% and 100% of reads

Randomly selected 70% and 80% of the total reads in pool 7 were used for detecting variants with GATK ploidy setting of 20. Ten rounds of randomization have been performed for selecting 70% and 80% of all reads. The estimated MAFs were based on allele counts of exome sequencing. In each SNV, the average MAF value of 10 rounds was used for MAF evaluation. The Y-axis shows the deviation between estimated MAF with WES and experimental validated MAF with genotyping. The X-axis shows each SNV for genotyping validation. The blue circles and red crosses represent estimating MAF with 70% and 80% of total reads respectively and the green triangles represent estimating MAF with total reads.

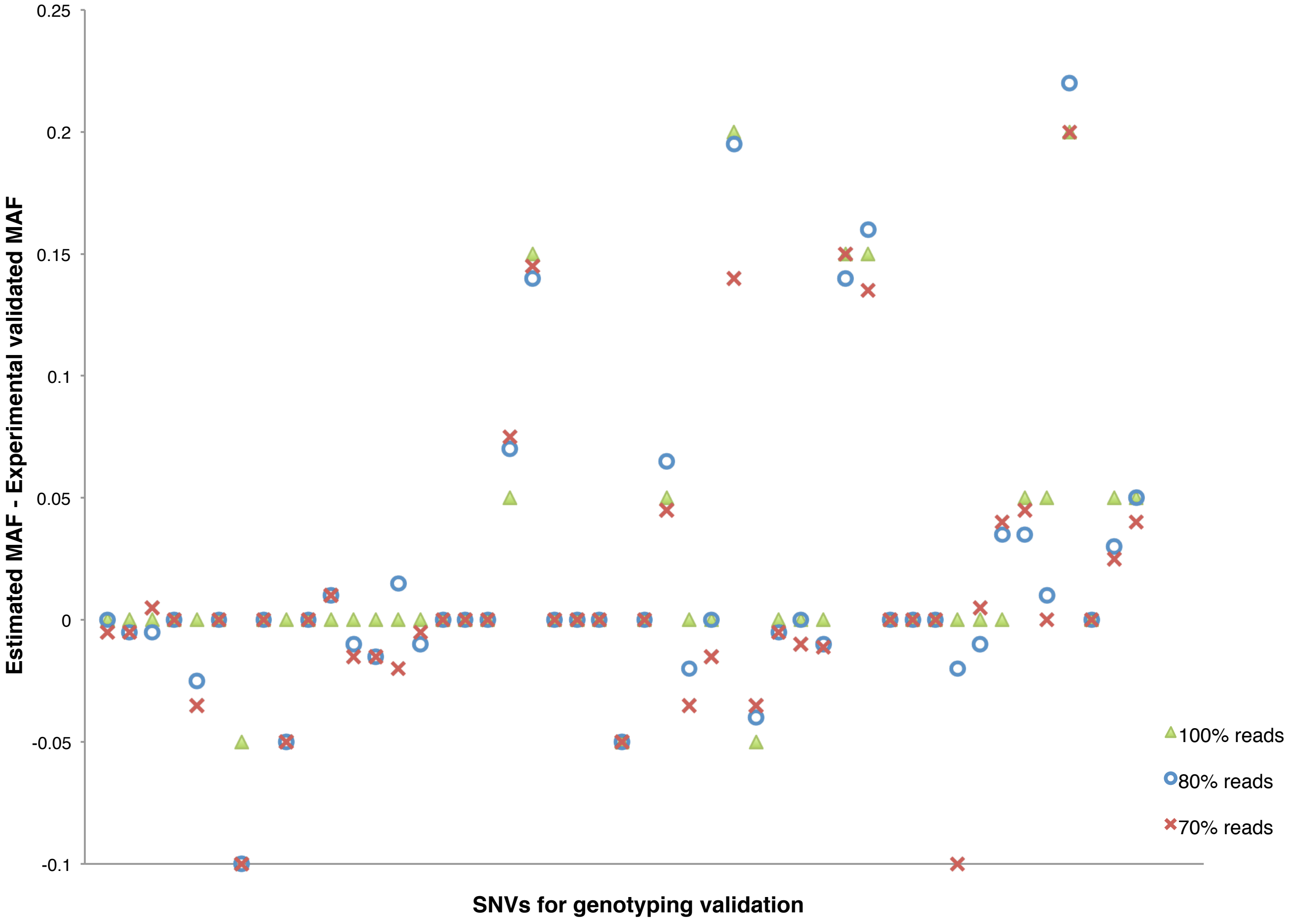

Figure S9. SNVs detected with different tools in the Bull Terrier whole genome sequencing

Red, blue and yellow circles represent the numbers of SNVs detected by Freebayes, GATK and SAMtools, respectively.

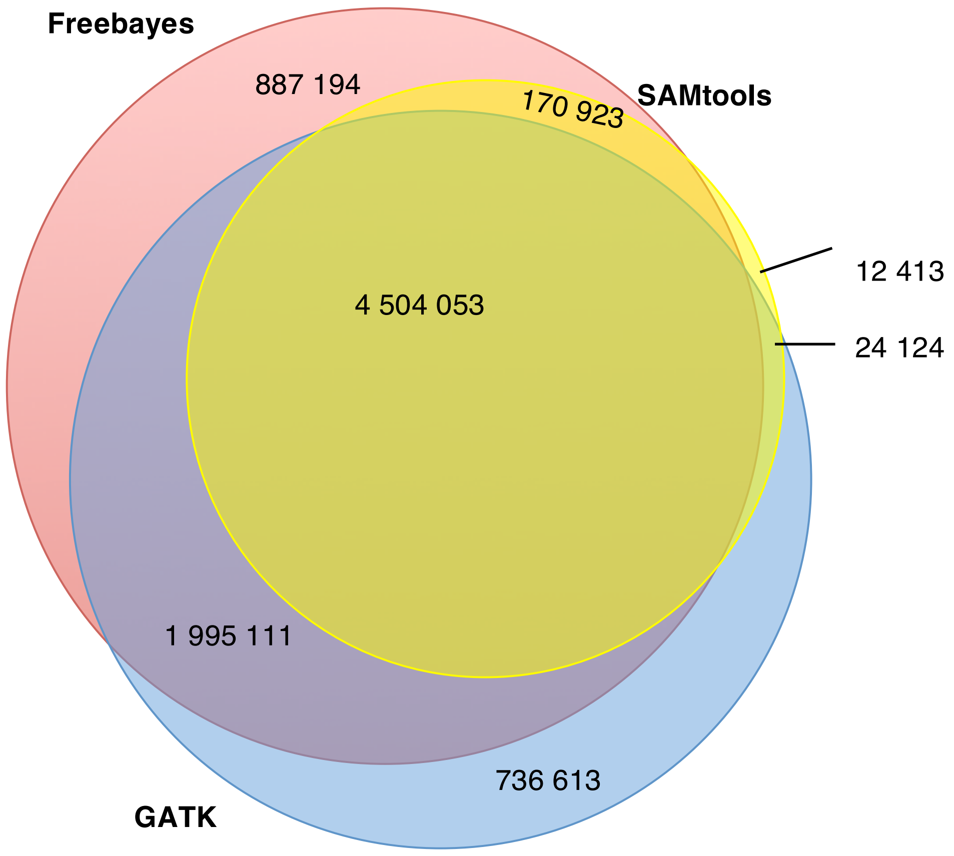

Figure S10. Distribution of allele difference between the WGS and the Illumina array including monomorphic markers
In total 164,868 SNP markers in the affected pool and 164,841 SNP markers in the unaffected pool were divided in two groups (MAF ≤ 5% and MAF > 5%), based on MAF in the Illumina array. The orange part represents the number of monomorphic sites detected in both platforms. The numbers in other colour labels = absolute value of allele difference between two platforms (allele count in WGS – allele count in the Illumina array).

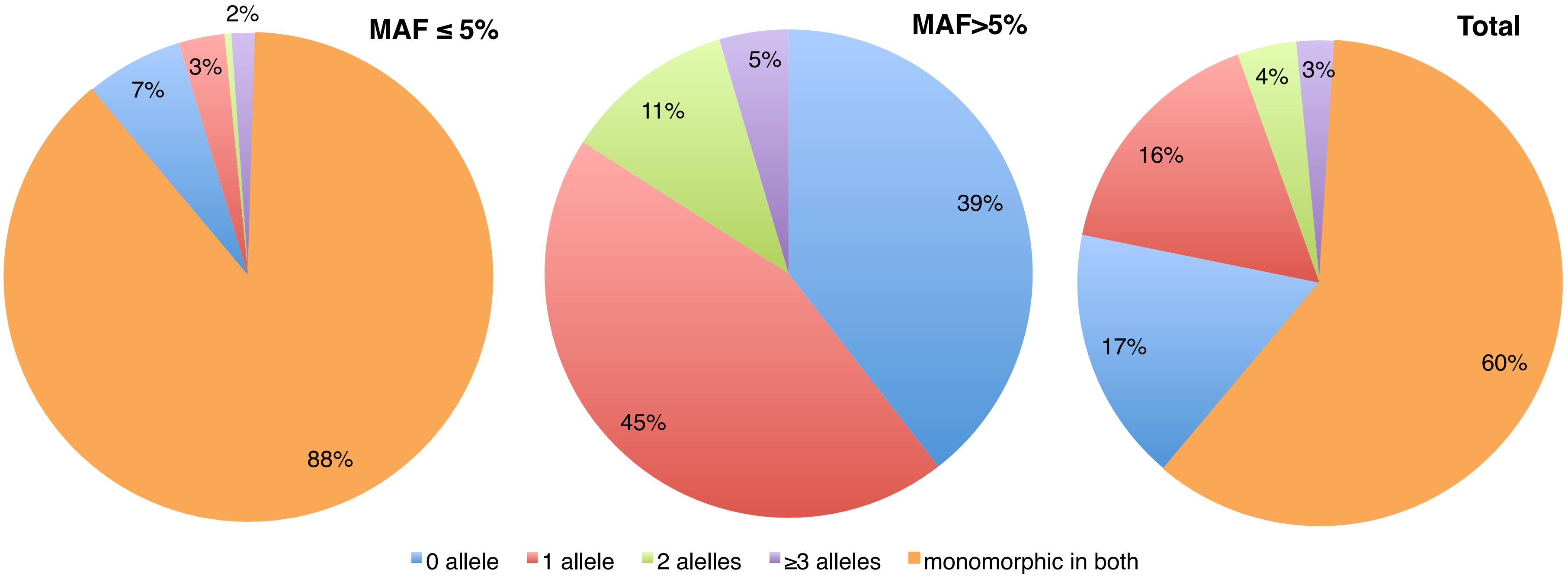

Supplement: Supplementary Information [file srep33256-s1.pdf]
